# Effect of the Memory Training for Recovery–Adolescent Intervention vs Treatment as Usual on Psychiatric Symptoms Among Adolescent Girls in Afghanistan: A Randomized Clinical Trial

**DOI:** 10.1001/jamanetworkopen.2023.6086

**Published:** 2023-03-30

**Authors:** Sayed Jafar Ahmadi, Laura Jobson, Zeinab Musavi, Sayed Rohullah Rezwani, Farshad Ahmad Amini, Arul Earnest, Nasratullah Samim, Sayed Ali Akbar Sarwary, Sayed Abbas Sarwary, Daniel McAvoy

**Affiliations:** 1 Bard College, Annandale on Hudson, New York; 2 Turner Institute for Brain and Mental Health and School of Psychological Sciences, Monash University, Melbourne, Victoria, Australia; 3 Behrawan Research and Psychology Services Organization, Kabul, Afghanistan; 4 Biostatistics Unit, Department of Epidemiology and Preventative Medicine, School of Public Health and Preventative Medicine, Monash University, Melbourne, Victoria, Australia; 5 Centre for Humanitarian Leadership, Deakin University, Melbourne, Victoria, Australia

## Abstract

**Importance:**

Adolescents who experience conflict in humanitarian contexts often have high levels of psychiatric distress but rarely have access to evidence-based interventions.

**Objective:**

To investigate the efficacy of Memory Training for Recovery–Adolescent (METRA) intervention in improving psychiatric symptoms among adolescent girls in Afghanistan.

**Design, Setting, and Participants:**

This randomized clinical trial included girls and young women aged 11 to 19 years with heightened psychiatric distress living in Kabul, Afghanistan, and was conducted as a parallel-group trial comparing METRA with treatment as usual (TAU), with a 3-month follow-up. Participants were randomized 2:1 to receive either METRA or TAU. The study occurred between November 2021 and March 2022 in Kabul. An intention-to-treat approach was used.

**Interventions:**

Participants assigned to METRA received a 10-session group-intervention comprised of 2 modules (module 1: memory specificity; module 2: trauma writing). The TAU group received 10 group adolescent health sessions. Interventions were delivered over 2 weeks.

**Main Outcomes and Measures:**

Primary outcome measures were self-reported posttraumatic stress disorder (PTSD) and depression symptoms after the intervention. Secondary outcomes were self-reported measures of anxiety, Afghan-cultural distress symptoms, and psychiatric difficulties. Assessments occurred at baseline, after modules 1 and 2, and at 3 months after treatment.

**Results:**

The 125 participants had a mean (SD) age of 15.96 (1.97) years. Overall sample size for primary analyses included 80 adolescents in the METRA group and 45 adolescents in TAU. Following the intention-to-treat principle, generalized estimating equations found that the METRA group had a 17.64-point decrease (95% CI, −20.38 to −14.91 points) in PTSD symptoms and a 6.73-point decrease (95% CI, −8.50 to −4.95 points) in depression symptoms, while the TAU group had a 3.34-point decrease (95% CI, −6.05 to −0.62 points) in PTSD symptoms and a 0.66-point increase (95% CI, −0.70 to 2.01 points) in depression symptoms, with the group × time interactions being significant (all *P* < .001). METRA participants had significantly greater reductions in anxiety, Afghan-cultural distress symptoms, and psychiatric difficulties than TAU participants. All improvements were maintained at 3-month follow-up. Dropout in the METRA group was 22.5% (18 participants) vs 8.9% for TAU (4 participants).

**Conclusions and Relevance:**

In this randomized clinical trial, those in the METRA group had significantly greater improvements in psychiatric symptoms relative to those in the TAU group. METRA appeared to be a feasible and effective intervention for adolescents in humanitarian contexts.

**Trial Registration:**

anzctr.org.au Identifier: ACTRN12621001160820

## Key Points

**Question:**

Can Memory Training for Recovery–Adolescent (METRA) improve psychiatric symptoms among adolescent girls affected by war in Afghanistan?

**Findings:**

In this randomized clinical trial of 125 adolescent girls with heightened psychiatric distress, those who received METRA had significantly greater reductions in posttraumatic stress disorder and depression symptoms than those allocated to treatment as usual, and these improvements were maintained at the 3-month follow-up.

**Meaning:**

The findings indicate that METRA is an effective, feasible, and acceptable treatment for symptoms of posttraumatic stress disorder and depression in adolescent girls affected by war in Afghanistan.

## Teaser

This randomized clinical trial investigates the efficacy of the Memory Training for Recovery–Adolescent (METRA) intervention in improving psychiatric symptoms among adolescent girls in Afghanistan.

## Introduction

Adolescents exposed to conflict in humanitarian settings in low- and middle-income countries (LMICs) experience concerning levels of psychiatric distress.^1,2^ While addressing these issues is a humanitarian priority,^3,4,5^ the needs of adolescents in these settings continue to receive insufficient attention in psychiatry.^6^ The Afghan humanitarian crisis is one of the world’s most complex and severe humanitarian emergencies.^7,8^ Afghanistan has one of the world’s youngest populations; adolescents account for approximately 26% of the population.^9^ The long history of armed conflict, poverty, and social injustice has impacted the mental health of Afghan youths, particularly girls.^9,10,11,12^ For instance, in a sample of 1011 Afghan adolescents, 18.0% and 23.9% met criteria for emotional concerns and posttraumatic stress disorder (PTSD), respectively, with girls being more than twice as likely to have a psychiatric disorder as boys.^10^

Compounding the issues facing Afghan adolescents, the United States and its allies left Afghanistan and the Taliban regained control in August 2021. The United Nations reported that “the needs of children of Afghanistan have never been greater”^13^ and “adolescents are struggling with anxieties and fears, in desperate need of mental health support.”^14^ Thus, questions rapidly emerged about the mental health of Afghan adolescents. A study conducted following these sociopolitical changes found, among a sample of 376 adolescents, approximately half met criteria for PTSD, depression, or anxiety,^15^ with more girls vs boys meeting criteria for PTSD (79.4% vs 31.0%) and depression (79.4% vs 26.4%).^15^ The developmental process of being an adolescent in Afghanistan can be very difficult.^9^

Addressing psychiatric concerns during adolescence is imperative,^4,5,16^ particularly as the well-being of adolescents and their ability to contribute to society is essential for the prosperity of Afghanistan.^17^ However, very few adolescents in LMICs, including Afghanistan, receive evidence-based interventions due to high costs, limited health services, and a shortage of skilled professionals.^8,18,19,20^ To meet these psychiatric needs, we proposed that Memory Training for Recovery–Adolescent (METRA), an evidence-based, low-intensity training, may improve adolescent mental health; specifically, PTSD and depression symptoms.

Those with depression and PTSD, including war-affected adolescents,^4^ exhibit certain autobiographical memory disruptions.^21,22,23^ METRA targets 2 memory disruptions. First, those with PTSD and depression have difficulties remembering specific personal events (eg, “I attended Adina’s party on Friday”) and instead provide overgeneral memories (OGM) (eg, “I attend parties every weekend”).^23^ OGM in adolescents is associated with ongoing psychiatric difficulties, which can persist into adulthood.^24^ OGM develops as a cognitive avoidance strategy in response to distressing memories^23^ and is associated with impaired problem-solving, avoidance, rumination, and difficulty accessing specific past and future information, processes integral to recovery from PTSD and depression.^16,22,23,24^ Targeting memory specificity improves PTSD and depression,^16,25,26^ and including a memory specificity training prior to trauma memory–focused interventions is proposed to have beneficial effects.^25^ Thus, module 1 of METRA targets memory specificity.^27^ Second, as trauma memories are often intrusive and distressing,^21^ evidence-based PTSD interventions target the trauma memory.^26^ Module 2 is writing for recovery, ie, written exposure training targeting the trauma memory.^28,29,30,31,32,33^ There is enormous untapped potential to improve the psychosocial functioning of war-affected adolescents by targeting these memory difficulties underpinning posttraumatic distress. Our pilot findings focusing on the individual modules of METRA have been promising.^16,25,29,33^ However, research has not investigated the efficacy, feasibility, and acceptability of METRA in a LMIC humanitarian setting.

This study conducted a randomized clinical trial (RCT) to investigate the efficacy, feasibility, and acceptability of METRA in addressing psychiatric concerns among adolescents in a humanitarian context. The study originally planned to recruit both boys and girls. However, after the change in government, it was identified by local nongovernment organizations (NGOs) that there was a particular unmet need regarding the psychiatric needs of girls. Thus, we focused on adolescent girls. The primary objective of the study was to investigate the efficacy of METRA in improving PTSD and depression symptoms in adolescents living in an LMIC humanitarian context. The secondary objectives were to investigate (1) the efficacy of METRA in improving general psychiatric symptoms (anxiety, Afghan idioms of distress, psychiatric difficulties); (2) whether improvements in symptomatology were maintained at 3-month follow-up; and (3) the feasibility and appropriateness of METRAs.

## Methods

### Trial Design

**Figure 1.**
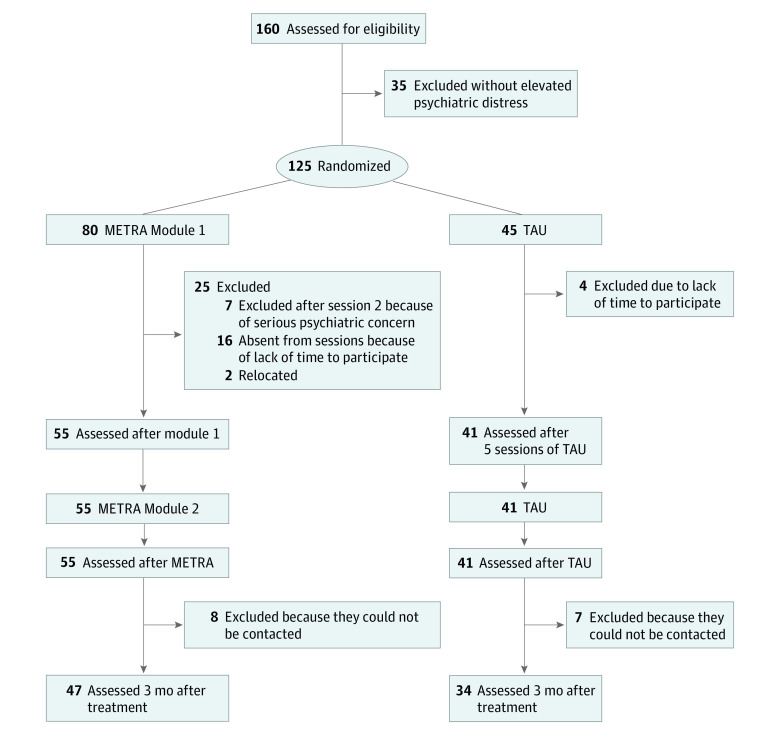
Flowchart of Participant Recruitment and Assessment METRA indicates Memory Training for Recovery–Adolescent; TAU, treatment as usual.

The trial was approved by Shaheed Prof. Rabbani Education University’s institutional review board and the Afghan Ministry of Health. This study followed the Consolidated Standards of Reporting Trials (CONSORT) reporting guideline. Written informed consent was obtained from adolescents, and verbal informed consent was obtained from their parents or guardians. The trial protocol (versions 1 and 2) is presented in Supplement 1. Due to the government change and subsequent security issues, several changes were made to the trial design prior to commencing recruitment and obtaining ethical approval but after first registering the study. These changes were updated in the trial registration and are highlighted in version 2 of the protocol. To examine our objectives, we used a parallel-group RCT comparing METRA with treatment as usual (TAU), with a 3-month follow-up. Participants were assessed in Pashto or Dari at baseline, after modules 1 and 2, and at 3-month follow-up. Figure 1 presents the study flow diagram.

### Participants

Two hundred girls were approached through a local government school and community organizations in Kabul and invited to participate. One hundred and sixty girls agreed to participate, and 125 girls aged 11 to 19 years met eligibility criteria and were randomly allocated to METRA or TAU. Eligibility criteria were as follows: Afghan girl aged 10 to 19 years with elevated psychiatric distress, defined as a score of greater than 30 on the Child Revised Impact of Event Scale–13 (CRIES-13)^34^ and/or a score greater than 12 on the Mood and Feeling Questionnaire–Short Form (MFQ-SF).^16^ Exclusion Criteria were as follows: high levels of suicidality; unmanaged psychosis or manic episodes in the past month; and presence of head trauma or organic brain damage as assessed by a clinical psychologist during a prestudy clinical interview.

A priori power analysis was undertaken using G*Power for depression and PTSD outcomes to detect a small to moderate interaction effect (*f* = 0.15)^16,29^ between group (METRA and TAU) and time (pretest and posttest) at α = .05. A sample size of 90 participants would achieve power greater than 0.80.

### Procedure

Enrolment began in November 2021, and data collection ended in March 2022. As secondary schools were closed to girls since August 2021, the assessments were conducted in a rented property in Kabul. Following baseline assessment, participants were randomized to METRA or TAU. Those allocated to METRA received module 1 (5 sessions delivered over 3 mornings and 2 afternoons within a week) and then, 4 days later, module 2 (5 daily sessions). METRA was conducted in a rented property in Kabul. The TAU group received 10 sessions of an adolescent health program delivered by a local NGO.

### Randomization and Blinding

After eligibility was confirmed and baseline data collected, the study coordinator randomly assigned participants in a 2:1 ratio to METRA or TAU, using a computer-generated randomization sequence. Following the change of government, there was increased insecurity, and most international agencies withdrew from Afghanistan. Thus, at the time of the study there were very few youth health interventions available that could be considered TAU. We changed our randomization approach and adopted an unequal randomization approach for resource and ethical reasons to maximize the number of girls in the treatment group. This unequal randomization approach has been used in previous trauma-focused RCTs, including those in LMICs.^35,36^ This decision should not have affected power for our statistical tests nor changed the scientific rigor of the study.^37^ We used consecutively numbered sealed opaque envelopes to conceal the allocation. The research coordinator in Kabul (independent of intervention delivery) monitored generation of the allocation sequence, participant enrolment, and assigning participants to interventions. Assessments were conducted by independent raters who had no therapeutic relationship with participants and were blind to aims and group allocation.

### Measures

#### Primary Outcomes

CRIES-13 is a 13-item self-report measure of PTSD symptoms.^34^ Items were rated on 6-point scales, with higher scores indicating greater PTSD symptoms.^34^ It has good psychometric properties^35^ and has been used with Afghan adolescents.^10,16,29^ Internal consistency was good (McDonald ω = 0.87).

MFQ-SF is a 13-item questionnaire assessing depression.^38^ Items were rated on 3-point scales with respect to the past 2 weeks. Items were summed to provide a total depression symptom score, with higher scores indicating greater depression severity. It has good psychometric properties^38^ and has been used with Afghan youth.^16^ Internal consistency was good (McDonald ω = 0.93).

#### Secondary Outcomes

Secondary outcomes were assessed using the Revised Children’s Manifest Anxiety Scale (anxiety),^39^ Strengths and Difficulties Questionnaire (psychiatric difficulties),^40^ and Afghan Symptom Checklist (culture-specific idioms of distress).^41,42^ All measures have been used with Afghan youth,^10,12,15,29^ and internal consistency was good (McDonald ω > 0.78) (eMethods in Supplement 2).

### Acceptability and Feasibility

We assessed feasibility of recruitment by determining the number of adolescents who were approached and agreed to participate in METRA. We assessed acceptability of intervention by measuring loss to follow-up. We determined acceptability of treatment based on the number of METRA sessions attended by adolescents. Following METRA, we conducted 10 interviews with adolescents who provided feedback on METRA.

### Interventions

#### TAU

A local NGO provided the course of intervention that they deemed appropriate. No specific instructions were given as to what TAU should entail, except not including elements specific to METRA. All adolescents in the TAU group were offered 10 sessions of an adolescent health group (8-10 adolescents/group) focused on mental health literacy, relationships, puberty, and maintaining physical and mental health. The program did not discuss trauma. The groups were facilitated by local NGO health staff. The number and timing of sessions was similar to that of METRA.

#### METRA

METRA is a manualized group training (8 adolescents/group) comprised of 2 modules, with both modules including five 1-hour sessions. Module 1 was based on Memory Specificity Training.^16,26,28^ Session 1 provided psycho-education, and participants practiced recalling specific memories in response to positive and neutral cues. Session 1 homework included generating a specific memory for 10 cues (positive and neutral). Session 2 included a summary of session 1, homework review, and further practice recalling memories in response to positive and neutral cues. Session 2 homework was the same as session 1. Session 3 was similar to session 2; however, participants now worked with negative cues. Session 4 involved exercises using negative and counterpart positive cues and discussions and exercises to promote metacognitive awareness. Session 5 included further practice and a summary of module 1.

Module 2 was a modified form of written exposure therapy and writing for recovery.^30,31,32,33,34,43,44^ Session 1 included a brief outline of the purpose of module 2. In the 5 sessions, adolescents wrote about their trauma for a full 30 minutes. They were encouraged to write about the details of the trauma(s) as they remember it now (including specifics of what happened, thoughts and feelings, worst aspects of the event, how the event had touched their life). After the 30 minutes, the facilitator asked adolescents to finish up, thanked them for their efforts, and ensured adolescents were ready to leave. Adolescents left their workbooks behind for the next session. Between sessions facilitators read the narratives to ensure participants had understood the task and were engaging appropriately.

### Facilitators and Treatment Fidelity

METRA was delivered by facilitators (community members with a health or education background) with minimal training; each facilitator received 8 hours of training (two 4-hour sessions). Facilitators received supervision from a clinical psychologist. A random 25% of the audio-recorded sessions were rated for manual adherence using a Therapist Adherence Scoring sheet. To reduce group contamination, participants were asked not to discuss the treatment with others.

### Statistical Analysis

Data were analyzed using Stata version 17 (StataCorp). Analyses were on intention-to-treat principle, with all randomized participants analyzed in their allocation condition. The first 3 objectives (PTSD and depression symptoms; anxiety, Afghan idioms of distress, and psychiatric difficulties; and changes at 3 months) were examined using a generalized estimating equation (GEE) model. We fit a model with a gaussian distribution, identity link, and an exchangeable correlation matrix. Of primary interest was the intervention × time interaction, which compared the levels of change over time in outcomes of the METRA and TAU groups.

## Results

### Group Characteristics

The 125 participants had a mean (SD) age of 15.96 (1.97) years; 80 were assigned to the METRA group and 45 to the TAU group. Participants in the METRA group had a mean (SD) age of 15.83 (2.07) years and had a mean (SD) of 8.16 (1.80) family members. Participants in the TAU group had a mean (SD) age of 15.65 (1.85) years and a mean (SD) of 8.59 (2.22) family members. Summary of baseline data for those who dropped out, those with adverse events, and those who completed the intervention is presented in the eTable in Supplement 2.

### Postintervention PTSD and Depression Symptomatology

**Figure 2.**
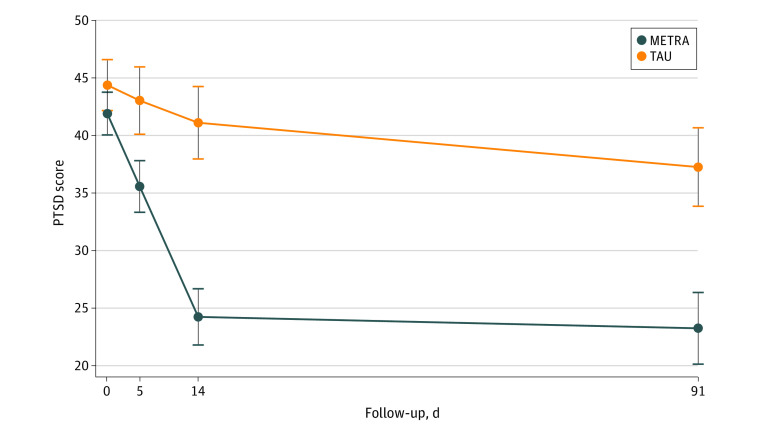
Marginal Means for Posttraumatic Stress Disorder (PTSD) Symptoms by Treatment Group and Follow-up Period Whiskers represent 95% CIs. METRA indicates Memory Training for Recovery–Adolescent; TAU, treatment as usual.

**Figure 3.**
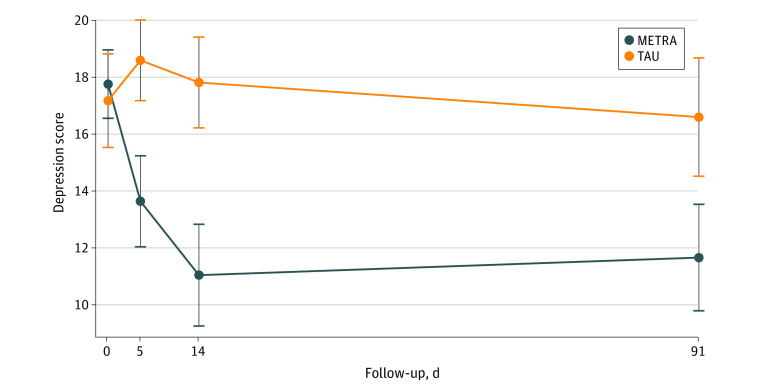
Marginal Means for Depression Symptoms by Treatment Group and Follow-up Period Whiskers represent 95% CIs. METRA indicates Memory Training for Recovery–Adolescent; TAU, treatment as usual.

Descriptive data for the outcomes of PTSD and depression symptoms are provided in Table 1 and Figures 2 and 3. GEE indicated that for both PTSD and depression symptoms the group × time interactions were significant. After the intervention, the METRA group had a 17.64-point decrease (95% CI, −20.38 to −14.91 points) in PTSD symptoms from baseline, while the TAU group had a 3.34-point decrease (95% CI, −6.05 to −0.62 points; *P* < .001). The METRA group had a 6.73-point decrease (95% CI, −8.50 to −4.95 points) in depression symptoms from baseline, while the TAU group had a 0.66-point increase (95% CI, −0.70 to 2.02 points; *P* < .001).

**Table 1.** Outcome Measures by Condition and Time Point for Treatment Outcomes

Outcome measure	Marginal mean (95% CI)			
	Baseline	After module 1	After intervention	Follow-up
**PTSD symptoms**				
METRA	41.93 (40.07-43.78)	35.59 (33.35-37.84)	24.27 (21.82-26.71)	23.27 (20.16-26.39)
TAU	44.40 (42.19-46.61)	43.06 (40.13-45.98)	41.13 (38.98-44.28)	37.28 (33.87-40.69)
**Depression symptoms**				
METRA	17.78 (16.56-18.97)	13.64 (12.03-15.24)	11.04 (9.24-12.83)	11.65 (9.78-13.53)
TAU	17.11 (15.44-18.78)	18.55 (16.80-20.28)	17.77 (16.02-19.49)	16.57 (14.66-18.48)
**SDQ difficulties**				
METRA	18.55 (17.38-19.72)	16.96 (15.56-18.36)	13.96 (12.48-15.45)	12.93 (11.09-14.78)
TAU	18.76 (17.35-20.16)	19.77 (18.42-21.11)	19.01 (17.80-20.22)	19.38 (17.75-21.01)
**Afghan-culture specific symptoms**				
METRA	49.58 (46.15-53.00)	37.61 (33.59-41.62)	31.93 (27.62-36.25)	35.20 (30.72-39.68)
TAU	49.36 (44.61-54.10)	51.29 (46.95-55.63)	47.78 (43.50-52.06)	46.97 (41.79-52.15)
**Anxiety**				
METRA	26.20 (25.30-27.10)	22.98 (21.92-24.03)	21.50 (20.26-22.74)	20.98 (19.21-22.75)
TAU	26.22 (25.15-27.30)	26.12 (24.70-27.55)	26.66 (25.44-27.88)	25.17 (23.67-26.68)

Abbreviations: METRA, Memory Training for Recovery–Adolescent; PTSD, posttraumatic stress disorder; SDQ, Strengths and Difficulties Questionnaire; TAU, treatment as usual.

### Postintervention General Psychiatric Symptoms

Descriptive data for outcome of general psychiatric symptoms are provided in Table 1 as well as eFigures 1, 2, and 3 in Supplement 2. After the intervention, the METRA group had a 4.70-point decrease (95% CI, −6.10 to −3.29 points) in anxiety symptoms from baseline, while the TAU group had a 0.43-point increase (95% CI, −0.82 to 1.67 points; *P* < .001). The METRA group had a 17.62-point decrease (95% CI −21.56 to −13.67 points) in Afghan-cultural distress symptoms, while the TAU group had a 1.52-point decrease (95% CI, −6.55 to 3.52 points; *P* < .001). The METRA group had a 4.59-point decrease (95% CI, −5.96 to −3.21 points) in psychiatric difficulties, while the TAU group had a 0.25-point increase (95% CI, −0.97 to 1.47 points; *P* < .001).

### Follow-up Analyses

At follow-up, compared with baseline, the METRA group had an 18.59-point decrease (95% CI, −21.85 to −15.32 points) in PTSD symptoms (TAU: −7.23 [95% CI, −10.49 to −3.97] points), 6.12-point decrease (95% CI, −7.94 to −4.29 points) in depression symptoms (TAU: −0.54 [95% CI, −2.93 to 1.85] points), 5.21-point decrease (95% CI, −7.06 to −3.36 points) in anxiety (TAU: −1.06 [95% CI, −2.60 to 0.48] points), 14.39-point decrease (95% CI, −18.11 to −10.66 points) in Afghan-cultural distress symptoms (TAU: −2.36 [95% CI, −7.69 to 2.97] points), and 5.62-point decrease (95% CI, −7.64 to −3.60 points) in psychiatric difficulties (TAU: 0.61 [95% CI, −1.03 to 2.26] points). All differences in group × time were significant (all *P* ≤ .001).

### Feasibility and Acceptability

All participants allocated to the METRA group commenced METRA. Eighteen participants (22.5%) allocated to METRA dropped out during module 1, as they did not have time to complete METRA or had moved and were unable to attend due to distance, while 4 participants (8.9%) dropped out of TAU, reporting that they did not have time; (χ^2^_1_ = 6.21; *P* = .01). Fifteen participants (METRA, 8; TAU, 7) were lost to follow-up; all participants had relocated for security reasons and could not be contacted. Qualitative feedback was broadly positive and is summarized in Table 2.

**Table 2.** Summary of Qualitative Feedback

Theme	Details
Headache reduction	Several adolescents reported that they had many headaches in the past, but METRA had helped them to have fewer headaches and the pain has become more bearable.
Memory improvement	Some participants mentioned that their memory has improved. In this regard, they were more satisfied with module 1.
Concentration improvement	Several participants noted that they now have better concentration at school and work.
Increased tolerance	Some participants noted that they now react less quickly and can tolerate and manage their anger. They mentioned that even their family had noticed this improvement, and for this reason, the family encouraged them to participate in METRA.
Reduction of sadness and depression	Several participants reported that they now feel happier after completing METRA.
Improving communication	Some participants noted that after METRA, they can communicate better with others and have fewer fights with family members at home.
Improved sleep	Several participants reported that they now fall asleep easily.
Improved decision-making	Several participants reported that they now have the ability to make better decisions.
Modules	The first model was more attractive to several participants. In module 2, there was some resistance to writing. And in the first sessions, participants cried at times and wanted to avoid writing. However, participants noted that they did not leave the sessions. They noted that they had learned in module 1 to write about their positive memories at home and used this skill between sessions in module 2 when they felt upset and sad, and it made them feel better.

Abbreviation: METRA, Memory Training for Recovery–Adolescent.

### Adverse Events

In session 2, 7 participants (8.8%) assigned to METRA were excluded from the study and referred to the local hospital, as they presented with serious psychiatric symptoms. These 7 adolescents had been particularly impacted by a recent terrorist attack and all closely witnessed the graphic death of a sister or friend. The discussion of trauma in METRA session 1 appeared to exacerbate symptoms. We conducted sensitivity analyses by assuming there were no changes in the outcomes for those 7 participants after the intervention and used their baseline outcomes to impute postintervention outcomes. A similar pattern of results was found (eResults in Supplement 2). For those who completed METRA, there were no important harms or unintended effects reported.

## Discussion

This study investigated METRA in addressing psychiatric concerns among adolescent girls in an LMIC humanitarian context. After the intervention, those adolescents allocated to METRA had fewer symptoms of PTSD, depression, anxiety, cultural distress, and psychiatric difficulties than those allocated to TAU. These improvements were maintained at 3-month follow-up. Acceptability of METRA was generally high. While dropout rates were significantly higher for METRA compared with TAU (22.5% vs 8.9%), the METRA dropout rate was similar to that observed in other low-intensity PTSD interventions^43^ and lower than that often observed in high-intensity PTSD interventions.^43^ All participants dropped out in the first 2 sessions, reporting that they did not have time or their families had moved. Seven adolescents who had personally witnessed a recent terrorist attack were unable to proceed with METRA, as the session 1 psycho-education appeared to exacerbate psychiatric symptoms. Thus, care is needed when selecting participants for METRA regarding recent exposure to terrorist attacks or conflict.

This RCT was conducted in the months following the Taliban regaining power and the United States and its allies leaving Afghanistan. These changes posed several challenges to conducting the RCT as planned, and modifications were made to accommodate the current situation. Due to security concerns, the planned 10 weekly sessions were administered over a fortnight; assessments were shortened; and we only recruited from Kabul as our second recruitment site (Herat) was deemed insecure. Nevertheless, METRA was still able to be delivered to groups of adolescents by those with limited training, and adolescents reported satisfaction with METRA. Improvements in secondary outcomes suggest that METRA may drive nonspecific psychiatric improvement. This suggests there is potential for METRA to be generalized to other humanitarian settings and in cases where adolescents have not received a specific diagnosis but are experiencing symptoms of heightened distress.

### Limitations

This study has limitations, including changes to the protocol reflecting sociopolitical changes. However, all changes occurred prior to recruitment commencing. Flexibility in RCT and intervention delivery is essential in humanitarian contexts and needs to be accommodated to address the concerning gaps in the literature. Second, we focused on adolescent girls and recruited from Kabul. Thus, generalizability may be limited. Third, the lack of long-term follow-up precludes us from knowing whether METRA treatment gains were maintained beyond 3 months after treatment. Additionally, while previous research has identified the efficacy of module 1 in improving PTSD and depression, researchers have noted that a subsequent module targeting the trauma memory may be beneficial.^26^ Our findings support these notions; while module 1 did significantly improve symptoms, there seemed to be greater improvements during module 2 (eResults in Supplement 2). Further research is needed to test mechanisms of change.

## Conclusions

In this RCT, we found that adolescent girls in Afghanistan receiving METRA had significantly greater improvements in psychiatric symptoms relative to those receiving TAU. METRA appeared a feasible and effective intervention for adolescents in a complex humanitarian context.

## Notes

Supplement 1.
Trial Protocol, Original and Revised

Supplement 2.
eMethods.

eResults.
**eTable.** Summary of Baseline Data for Those Who Dropped Out, Those Excluded Due to Adverse Events, and Those Who Completed METRA and TAU **eFigure 1.** Marginal Means for Anxiety Symptoms by Treatment Group and Follow-up Period **eFigure 2.** Marginal Means for Afghan-Cultural Distress Symptoms by Treatment Group and Follow-up Period **eFigure 3.** Marginal Means for Psychiatric Difficulties (Strengths and Difficulties Questionnaire) by Treatment Group and Follow-up Period

Supplement 3.
Data Sharing Statement
