# Supplementary material for: Effect of the Memory Training for Recovery–Adolescent Intervention vs Treatment as Usual on Psychiatric Symptoms Among Adolescent Girls in Afghanistan: A Randomized Clinical Trial
Source: JAMA Netw Open. 2023 Mar 30;6(3):e236086. doi: 10.1001/jamanetworkopen.2023.6086 (PMC10064255; doi:10.1001/jamanetworkopen.2023.6086)
Supplement: Supplement 1. — Trial Protocol, Original and Revised [file jamanetwopen-e236086-s001.pdf]

Randomized Clinical Trial to evaluate efficacy, feasibility and appropriateness of MEmory Training for Recovery- Adolescent (METRA) among war-affected adolescents: A structured summary of a study protocol for a randomized controlled trial – Version 1

Abstract

Background: Adolescent refugees and war-affected youth in low- and middle-income countries (LMICs) generally do not receive evidence-based psychological interventions, as most interventions are complex, require specialist knowledge, and are prohibitively expensive. This study aims to investigate the efficacy of MEmory Training for Recovery-Adolescent (METRA) in improving psychological symptoms (posttraumatic stress disorder, depression) in war-affected adolescents delivered in LMIC humanitarian contexts. Our secondary aims are to examine the feasibility and appropriateness of METRA, explore the mechanisms mediating treatment effects, and to include a cost-analysis of METRA in LMIC humanitarian contexts.

Methods: We will use mixed methods to assess feasibility, appropriateness, and efficacy. The project will also include an embedded mechanism study and a cost-analysis component.

Discussion: Research outputs include an evaluation of METRA, an innovative, low-intensity, freely-available intervention that can be delivered by individuals with minimal training in humanitarian contexts. We anticipate that refugee and war-affected adolescents who experience high levels of psychological distress will benefit from this research.

Trial registration: We have submitted our trial for registration on the Australian New Zealand Clinical Trials Registry.

Keywords

Adolescent; Refugee; War-affected; Memory Training for Recovery; Trauma; Depression

Administrative information

The trial is funded by R2HC 2019 ELRHA funding (44708).

Title {1}	Randomized Clinical Trial to evaluate efficacy, feasibility and appropriateness of MEmory Training for Recovery- Adolescent (METRA) among war-affected adolescents: a structured summary of a study protocol for a randomized controlled trial
Trial registration {2a and 2b}.	Australian New Zealand Clinical Trials Registry (Trial Id: ACTRN12621001160820)
Protocol version {3}	July 2021. Version 1.
Funding {4}	The trial is funded by a Research for Health in Humanitarian Crises Grant (ELRHA) (44708). Funders: UKAID, Wellcome Trust, National Institute for Health Research
Author details {5a}	1) Associate Professor Laura Jobson – School of Psychological Sciences and Turner Institute for Brain and Mental Health, Monash University, Melbourne Australia<br>2) Dr Sayed Jafar Ahmadi - Shaheed Prof. Rabbani Education University, Kabul, Afghanistan<br>3) Zainab Mosavi – Behrawan Research and Psychological Services Organization, Kabul, Afghanistan<br>4) Sayed Rohollah Rezvani – Department of Psychology, University of Isfahan, Isfahan, Iran<br>5) Associate Professor Arul Earnest – Biostatistics Unit, School of Public Health and Preventive Medicine, Monash University, Melbourne, Australia<br>6) Dr Daniel McAvoy – Centre for Humanitarian Leadership, Deakin University, Melbourne Australia
Name and contact information for the trial sponsor {5b}	Monash University

Role of sponsor {5c}	N/A

Introduction

Background and rationale {6a}

War-affected and refugee adolescents in low- and middle-income countries (LMICs) experience concerning levels of psychological distress, including posttraumatic stress disorder (PTSD), depression, and anxiety (Attanayake, McKay, Joffres, Singh, Burkle, & Mills, 2009; Blackmore et al., 2020; Morina, von Lersner, & Prigerson, 2011; Tyrer & Fazel, 2014). Afghanistan has endured a long-history of armed conflict, poverty, and social injustice, which has significantly impacted on the mental health of Afghan youth (Panter-Brick, Eggerman, Gonzalez, & Safdar, 2009; Panter-Brick, Goodman, Tol, & Eggerman, 2011). Research has shown high levels of distress and poor mental health among Afghan youth, including PTSD, depression, anxiety and somatoform symptoms (Alemi et al., 2018). Indeed, in a recent study we found that all Afghan adolescent refugees recruited for our research were experiencing concerning levels of PTSD and depression (Mirabolfathi, Schweizer, Moradi, & Jobson, 2019). These symptoms severely affect an adolescent's life, including long-lasting cognitive, emotional, social and academic/vocational problems (Neshat-Doost, Yule, Kalantari, Rezvani, Dyregrov, & Jobson, 2013). Addressing psychological concerns in the critical developmental period of adolescence is imperative (Neshat-Doost et al., 2013; The Economist, 2019). However, very few refugee and war-affected adolescents in LMICs receive evidence-based interventions, due to high costs, limited mental health services and a shortage of skilled professionals (Juengsiragulwit, 2015; Zia, Afkhami, Tavakoli, Neshat-Doost, & Jobson, in press; Kohrt et al., 2018). The needs of adolescents in LMICs have not received sufficient attention in psychology and psychiatry research (Frontiers in Psychiatry, 2019). In order to meet these identified needs, we propose that MEmory Training for Recovery-Adolescent (METRA), a low-intensity, accessible and scalable evidence-based intervention, will improve adolescent mental health (specifically depression and PTSD), .

Trauma and Memory

Those suffering from depression and PTSD, including adolescent refugees and war-affected youth

(Neshat-Doost et al., 2013), exhibit certain disruptions in their autobiographical remembering (i.e., remembering personal experiences from one's life) (Brewin, 2011; Dalgleish & Werner-Seidler, 2014). Trauma memories are often intrusive and distressing (Brewin, 2011) and PTSD sufferers have considerable difficulties remembering specific events from their lives (e.g., "I attended Adina's party on Friday"). Instead, they provide overgeneral memories (OGM) of events ("I was attending a party every weekend") (Williams et al., 2007). OGM develops as a cognitive avoidance strategy in response to the distressing memories one may experience (Williams et al., 2007).

OGM is problematic as it is associated with impaired social problem-solving, cognitive avoidance, rumination, impairments in executive control, difficulty imagining specific events in the future (Kleim, Graham, Fihosy, Stott, & Ehlers, 2014; Sutherland & Bryant, 2008; Williams et al., 2007), and difficulty accessing specific information about the past, which interferes with the ability to update distressing memories (Moradi, Moshirpanahi, Parhon, Mirzaei, Dalgleish, & Jobson, 2014; Williams et al., 2007). Each of these processes are integral to recovery from PTSD and depression (Moradi et al., 2014; Neshat-Doost et al., 2013). Not surprisingly, an OGM retrieval style in adolescents has been found to predict on-going psychological difficulties, which can persist well into adulthood affecting social functioning and academic/vocational attainment (Hitchcock, Nixon, & Weber, 2014).

There is enormous untapped potential to improve the psychological functioning of refugee and war-affected adolescents by targeting these memory difficulties that underpin posttraumatic psychological distress. In response to this, we propose that MEmory Training for Recovery-Adolescents (METRA), a novel intervention for use in humanitarian contexts (Moradi et al., 2014; Neshat-Doost et al., 2013), may have potential in improving the psychological adjustment of adolescents.

Feasibility and Piloting of METRA Modules

METRA is an evidence-based, low-intensity, easily disseminable, transdiagnostic training package. It is comprised of two modules that target major cognitive features underpinning posttrauma psychological difficulties experienced by adolescent refugees; OGM and trauma/distressing memories.

Module 1 is a memory specificity training that teaches adolescents to recall specific positive, negative and neutral everyday memories from their lives (targets OGM) (Raes, Williams, &

Hermans, 2009). Module 2 is a writing for recovery, written exposure training (e.g., Kalantari, Yule, Dyregrov, Neshat-Doost, & Ahmadi, 2012; Sloan et al., 2018; Sloan & Marx, 2019) targeting distressing and trauma memories. Adolescents are supported in writing about their past including thoughts and feelings. The results of our pilot research, investigating the efficacy of the components of METRA, have been promising in the treatment of depression and PTSD in refugee adolescents and war veterans in LMICs (see Ahmadi, Kajbaf, Neshat Doost, Dalgleish, Jobson, & Mosavi, 2018; Kalantari et al., 2012; Moradi et al., 2014; Neshat-Doost et al., 2013). Furthermore, our studies have shown preliminary support that these modules were feasible in humanitarian settings, the training was well received by participants and organizations, and adolescents reported satisfaction and were motivated (Ahmadi et al., 2018; Kalantari et al., 2012; Moradi et al., 2014; Neshat-Doost et al., 2013).

METRA aligns with humanitarian initiatives aiming to deliver more accessible, low-resource (i.e., group-based, unskilled facilitator) psychosocial evidence-based interventions (Elrha, 2015). METRA, a low-intensity intervention, seems feasible in humanitarian settings and has potential for reducing PTSD and depression symptoms in refugee adolescents. There is a need to better understand the mechanisms driving the therapeutic effects of METRA and the costs associated with implementing METRA in humanitarian contexts. This project addresses identified humanitarian mental health research gaps (Elrha, 2015) by investigating the efficacy and feasibility of scaling-up a low-intensity modular transdiagnostic psychosocial interventions for adolescents. It also includes qualitative methods to examine appropriateness and acceptability of METRA and a health-economic component to undertake cost-analysis of the intervention.

Objectives {7}

Primary Objectives

1) Investigate the efficacy of METRA in improving psychological symptoms (posttraumatic stress disorder, depression) in adolescents living in refugee-like and humanitarian situations.

Secondary Objectives

2) Investigate the feasibility and appropriateness of METRA for adolescents living in refugee-like and humanitarian situations delivered in LMIC humanitarian contexts.

3) Examine the mechanisms mediating treatment effects.

4) Undertake a cost-analysis of METRA in LMIC humanitarian contexts.

Trial design {8}

This is a randomised controlled trial (RCT) comparing METRA to treatment as usual (TAU), with an embedded qualitative component. Participants will be assessed; 1) at baseline, 2) at post-Module 1, 3) at post-Module 2, and 4) at 6-month follow-up. The first three assessments are face-to-face assessment and will include all measures. The follow-up assessments will be conducted by phone/skype/zoom and will include the primary and secondary outcomes. Assessments will be conducted by independent raters who have no therapeutic relationship with participants and are blind to condition.

Methods: Participants, interventions and outcomes

Study setting {9}

The Afghan humanitarian crisis has been identified as one of the world's most complex, severe and protracted humanitarian emergencies, with no sign of abatement (UNHCR, 2017). Afghanistan has one of the world's youngest populations, with adolescents accounting for around 26 per cent of the population (UNICEF, 2019a). The developmental process of growing up and being an adolescent in Afghanistan is very difficult (UNICEF, 2019a). Adolescents in Afghanistan are affected everyday by conflict and violence, on-going insecurity, and periodic natural disasters, which can impede development (UNICEF, 2019a). Compounding this, Afghan adolescents have poor access to healthcare, education, nutrition and have few livelihood opportunities, which can have long-term impacts on their mental and physical health (UNICEF, 2019a). Unsurprisingly, then, many Afghan adolescents are experiencing concerning levels of psychological distress (Ashrafi, 2019; Pedneault, 2019; UNOCHA, 2017; UNHCR, 2019).

There are around 2.6 million displaced Afghans, half of whom are under the age of 18 (UNHCR, 2019). Since 2020 many Afghan refugees are returning to Afghanistan, with many returnees and internally-displaced peoples residing in Herat (third largest city in Afghanistan situated near the border of Iran) and Kabul (Afghan capital) (AFP, 2020; Amnesty, 2021; UNHCR, 2020). The focus on sustainable return and reintegration of Afghan refugees, alongside the COVID-19 pandemic, has meant that many Afghan adolescents are returning to Afghanistan, reducing their access to essential mental health services (AFP, 2020; HAMI, 2013; UNOCHA, 2017). The conflict in Afghanistan has resulted in the closure and destruction of health facilities. Conflict and insecurity continue to impact negatively on adolescent returnee's health; it is estimated that around 2 million children/adolescents will require humanitarian health assistance but face limited access to services (UNOCHA, 2017). Thus, Afghanistan context has been categorised as complex, very high crisis and impact, and with extreme humanitarian access constraints (ACAPS, 2019; HAMI, 2013).

This study will take place in two settings within Afghanistan – Kabul and Herat. Both of these settings have been identified as a high-risk complex humanitarian crisis (ACAPS, 2019; United Nations Office for the Coordination of Humanitarian Affairs, 2017, 2018; UN Children's Fund, 2019). Ensuring the mental health of Afghan adolescents is essential for the stability, security and prosperity of Afghanistan (UNHCR, 2018).

Eligibility criteria {10}

Afghan refugee/returnee/war-affected adolescents aged 10-19 years with elevated psychological distress. This age range aligns with our pilot work and definitions of 'adolescent' (UNICEF, 2019b; WHO, 2019). As in our pilot studies, elevated psychological distress is defined as >30 on the Persian Child Revised Impact of Event Scale-13 (Child Outcomes Research Consortium, 2019) and/or >12 on the Persian Mood and Feeling Questionnaire – Short form (Neshat-Doost et al., 2006). Participants will be recruited in Herat and Kabul. We will aim to recruit 300 participants (150 per site). Exclusion Criteria: a) high levels of suicidality, b) unmanaged psychosis/manic episodes in past month, and c) presence of head trauma/organic brain damage.

Who will take informed consent? {26a}

Researchers will gain informed consent from the adolescents and their guardians.

Additional consent provisions for collection and use of participant data and biological specimens {26b}

N/A

Interventions

Explanation for the choice of comparators {6b}

Treatment as usual (TAU): Local NGOs will provide the course of intervention that they deem appropriate. No specific instructions will be given as to what TAU should entail, except not including elements specific to METRA. TAU will be documented ensuring understanding of the duration, frequency and type of treatment administered.

Intervention description {11a}

METRA: Module 1: Memory specificity training (Ahmadi et al., 2018; Moradi et al., 2014; Neshat-Doost et al., 2013; Raes et al., 2009; Martens et al., 2019) is a manualized training delivered over

five 60-minute sessions to groups of 6-8 adolescents. MEST aims to enhance memory specificity through practice. Session 1 provides psycho-education about mental health and memory. Participants practice recalling memories in response to positive and neutral cues, with support from the group facilitator. Attention is paid to the contextual, spatio-temporal and sensory-perceptual details of the memories (Raes et al., 2009). Participants' responses are discussed in the group. At the end of the session, homework exercises are introduced; for 10 cues (positive and neutral) participants need to generate a specific memory and are instructed to write down a 'specific memory of the day' every evening of the coming week (Raes et al., 2009). Session 2 starts with a brief summary of Session 1, the homework exercises are discussed and the Session then follows the same format as Session 1, with further practice focusing on recalling memories in response to positive and neutral cues. At the end of Session 2, the homework is explained; participants need to generate two different specific memories for 10 cues (positive and neutral) and write down two different 'specific memories of the day' every evening of the coming week (Raes et al., 2009). Session 3 is very similar to Session 2. However, in Session 3, participants also need to work with negative cues. The homework exercises are similar to those outlined in Session 2, but now also include negative cues. Session 4 involves further exercises using negative and ('counterpart') positive cues. It is also explained how overgeneral thinking can be addressed by recalling a single specific experience and examples are discussed to promote metacognitive awareness of when participants are starting to shift to unspecific thinking or more general retrieval (Raes et al., 2009). Session 5 includes further practice and a summary of Module 1. Module 1 focuses on everyday remembering.

Module 2: Writing for Recovery is a written exposure training involves five sessions (Kalantari et al., 2012; Sloan et al., 2018). In the first session the purpose of Module 2 is outlined. In the following sessions, the facilitator simply reads the instructions and the participant completes the writing task; writing about their trauma including thoughts and feelings. After 30 minutes, the facilitator instructs the participants to stop writing.

Criteria for discontinuing or modifying allocated interventions {11b}

Discontinue trial if participants report significant distress or a significant proportion of participants report significant increase in symptomatology. This will be based on the reports and observations of the facilitators and decisions to continue/discontinue will be made by the trial monitoring body.

Strategies to improve adherence to interventions {11c}

Group facilitators will be trained and receive supervision. A random 25% of the audio-recorded treatment sessions will be rated for manual adherence. To reduce group contamination, participants will be requested to not discuss treatment with others.

Relevant concomitant care permitted or prohibited during the trial {11d}

N/A

Provisions for post-trial care {30}

Referrals to local mental health services and NGOs will be made for participants requiring further psychological care.

Outcomes {12}

Primary Outcomes: self-reported symptoms of PTSD (Child Outcomes Research Consortium, 2019) and depression (Neshat-Doost et al., 2006). We will assess statistical significance of change in the levels of PTSD and depression symptoms in the intervention and control groups.
Secondary Outcomes: Afghan specific symptoms (Miller et al., 2006; Rasmussen et al., 2014); anxiety (Reynolds & Richmond, 1978); and internalising/externalising problems (Alavi et al., 2009).
Process Measures: (parallel forms) memory specificity (Williams et al., 2007); rumination (Abela, Vanderbilt, & Rochon, 2004); and cognitive avoidance (Sexton & Dugas, 2008).
Qualitative data: Interviews with adolescents in the METRA arm of the trial will be conducted to examine appropriateness and feasibility of METRA.

The measures and interviews will be conducted in Pashto and Dari.

Participant timeline {13}

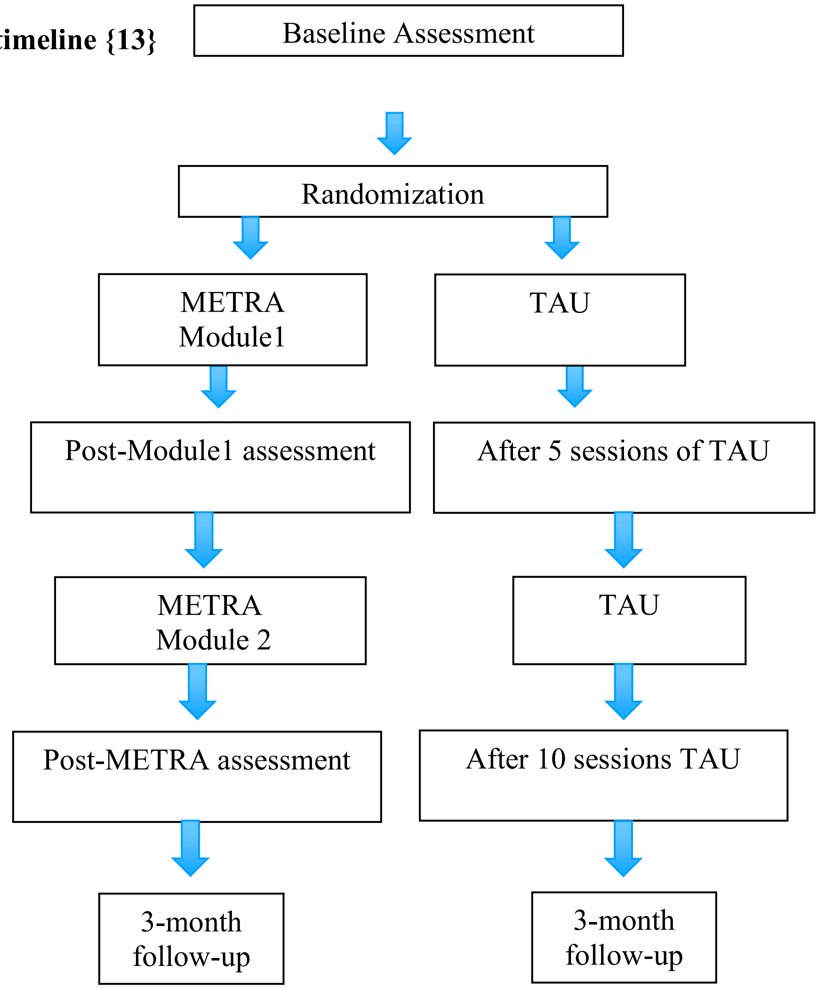

Figure 1: Flowchart of participant recruitment and assessment

Sample size {14}

A priori power analysis was undertaken using G*Power for depression and PTSD outcomes to detect a small to moderate interaction effect (f=0.15)[18,33] between group (intervention, control) and time (pre-test, post-test) at an alpha-level of 0.05. A sample size of 90 participants would achieve power >0.80. The sample size estimate was re-adjusted to allow for attrition and we aim to recruit >100 participants in both sites (Kabul and Herat).

Recruitment {15}

Participants will be recruited through local non-government agencies and schools in Herat and Kabul.

Assignment of interventions: allocation

Sequence generation {16a}

Following baseline assessment, sequential participants will be randomized to METRA or TAU.

Concealment mechanism {16b}

We will use consecutively numbered sealed opaque envelopes to conceal the allocation.

Implementation {16c}

The research team will oversee generation of the allocation sequence, participant enrollment, and assigning participants to interventions.

Assignment of interventions: Blinding

Who will be blinded {17a}

Outcome assessors will be blind to hypotheses and group allocation. Group facilitators will be blind to hypotheses.

Procedure for unblinding if needed {17b}

Not applicable

Data collection and management

Plans for assessment and collection of outcomes {18a}

Data will be collected at; 1) baseline, 2) post-Module 1, 3) post-Module 2, and 4) 6-month follow-up. Assessments will be conducted by independent raters who have no therapeutic relationship with participants and are blind to condition. All assessors will be trained and supervised in assessment procedures. All questionnaires have good reliability and validity and have been previously used cross-culturally.

Plans to promote participant retention and complete follow-up {18b}

Participants will be contacted using a variety of media, including phone, email and social media to promote retention and follow-up completion.

Data management {19}

Data will be coded, entered and stored in Kabul and Herat on password protected computer systems. Anonymized data will be shared amongst the research team.

Confidentiality {27}

Personal information about potential and enrolled participants will only be collected, shared, maintained and monitored by the research teams at each site. De-identified data will be shared between the team.

Plans for collection, laboratory evaluation and storage of biological specimens for genetic or molecular analysis in this trial/future use {33}

N/A

Statistical methods

Statistical methods for primary and secondary outcomes {20a}

All primary analyses will be on intent-to-treat principle, with all randomized participants analyzed in their allocation condition.

Objective 1 will be examined using linear mixed effects models with intervention type, time, and intervention by time interaction as fixed factors. Repeated assessments of individuals will be modelled as random intercept. Of primary interest will be the intervention by time interaction, which will compare the levels of change over time in outcomes of the METRA and TAU groups. Between-condition effect sizes (Cohen d) will be calculated and interpreted using guidelines from Cohen. Levels of significance will be set at $\alpha<.05$ and hypothesis tests will be 2-sided.. Our primary analysis will use data from both sites. Supplementary analyses include separate analyses being conducted for each site. Objective 2: Thematic analysis of qualitative data (Atkins et al., 2017; Braun & Clark, 2006; Marks & Yardley, 2004) will assess feasibility and appropriateness.

Objective 3: Mediational analyses will be carried out using regression-based approach outlined by McKinnon et al. (2007). This will assess mechanisms of change (memory specificity, rumination, avoidance).

Objective 4: We will look at the difference in outcomes and costs between METRA and TAU, allowing us to explore the cost per point change in symptoms for both. Difference in outcomes will be assessed by examining point changes in PTSD and depression symptoms based on our outcome scales. For costs, we will use bottom-up costing of both METRA and TAU whereby we identify the costs of all inputs (e.g., staff time: X minutes at $X salary per minute, X rooms at $X per room, exclusive of research-related costs) and sum them. We can then divide total costs by total point changes for each intervention and compare them. We will also compare total and per patient METRA program costs with existing mental health budgets in humanitarian contexts to give an indication of whether implementing METRA can be affordable in humanitarian settings.

Interim analyses {21b}

Not applicable

Methods for additional analyses (e.g. subgroup analyses) {20b}

Exploratory Analyses: Potential moderators (age, gender, trauma exposure, location, refugee experience) of intervention response will be explored with linear mixed models.

Methods in analysis to handle protocol non-adherence and any statistical methods to handle missing data {20c}

N/A

Plans to give access to the full protocol, participant level-data and statistical code {31c}

N/A

Oversight and monitoring

Composition of the coordinating centre and trial steering committee {5d}

The Trial Steering Committee will provide overall supervision of the project. It will include independent members who will monitor trial progress, functioning of the partnership and ensure local and community involvement.

Composition of the data monitoring committee, its role and reporting structure {21a}

Not applicable

Adverse event reporting and harms {22}

Should an adverse effect be reported, the Chief Investigators will be notified immediately and a plan to resolve the serious adverse effect will be formulated. Depending on the nature of the adverse event, the Chief Investigators and research team will determine the appropriate course of action and then the research team will discuss the situation fully with the participant. A complete list of adverse effects, the steps taken to resolve them, and the results of those steps, will be reported to the Ethics Review Committee, Trial Steering Committee, Kabul University and Behrawan monthly, while serious adverse effects will be reported within one business day.

Frequency and plans for auditing trial conduct {23}

The Trial Steering Committee will provide overall supervision of the project.

Plans for communicating important protocol amendments to relevant parties (e.g. trial participants, ethical committees) {25}

We will report changes to the ethic committees and trial registry.

Dissemination plans {31a}

The results will be published in peer-reviewed journal articles. There will also be an Engagement and Communication Strategy that will ensure key local (Afghanistan) and international stakeholders are informed about the findings.

Discussion

The complex humanitarian context of Herat and Kabul present operational challenges, as several of the universities are in the process of re-establishing themselves. However, our collaborations are well-established and we will follow the research protocols that were employed in our pilot work in this region.

Trial status

Protocol version number 1; July 2021.

Abbreviations

PTSD: Posttraumatic stress disorder

METRA: MEmory Training for Recovery – Adolescents

Declarations

Acknowledgements : N/A

Authors' contributions {31b}

LJ, S.A., A.R.M., H.N-D, V.M. are the Chief Investigators; they conceived the study, led the proposal and protocol development. FA, ZM, SR and DM contributed to study design and to development of the proposal. KV is the lead trial health economist and LB and AE are the lead trial biostatisticians. All authors read and approved the final manuscript.

Funding {4}

The trial is funded by R2HC 2019 ELRHA funding (44708).

Availability of data and materials {29}

The research team will have the final dataset and this dataset will be available by contacting the

researchers.

Ethics approval and consent to participate {24}

We will obtain ethical approval for the study from the Ministry of Health, Afghanistan.

Consent for publication {32}

Not applicable.

Competing interests {28}

The principal investigators have no competing interests.

References

Abela, J. R. Z., Vanderbilt, E., & Rochon, A. (2004). A test of the integration of the response styles and social support theories of depression in third and seventh grade children. *Journal of Social and Clinical Psychology, 23(5),* 653-674. doi: 10.1521/jscp.23.5.653.50752

ACAPS (2019). Crisis updates: Afghanistan. (https://www.acaps.org/country/afghanistan/crisis/complex-crisis)

AFP (2020) *Coronavirus: Nearly 70 000 Afghans return home after fleeing virus hit Iran*.

Ahmadi, S. J., Kajbaf, M. B., Neshat Doost, H. T., Dalgleish, T., Jobson, L., Mosavi, Z. (2018). The efficacy of memory specificity training in improving symptoms of post-traumatic stress disorder in bereaved Afghan adolescents. *Intervention, 16*, 243-248.

Alavi, Md & Mohammadi, Mohammad-Reza & Md, J & Tehranidoost, M & Shahrivar, Zahra & Saadat, Soheil. (2009). The Farsi version of the strength and difficulties questionnaire self report form: The normative data and scale properties. *Iranian Journal of Child Neurology. 3*.

Alemi, Q., Stempel, C., Koga, P.M. *et al.* Risk and protective factors associated with the mental health of young adults in Kabul, Afghanistan. *BMC Psychiatry 18,* 71 (2018). https://doi.org/10.1186/s12888-018-1648-4

Amnesty (2021, March 30). Afghanistan: Country's four million internally displaced need urgent support amid pandemic. https://www.amnesty.org/en/latest/news/2021/03/afghanistan-countrys-four-million-internally-displaced-need-urgent-support-amid-pandemic/

Afghanistan: Country's four million internally displaced need urgent support amid pandemic

Ashrafi, F. (2019). Fatemeh Ashrafi, the director of HAMI "Iran's immigrant politics is based on the Islamic brotherhood and understanding". http://hamiorg.org/en/?p=3609

Atkins, L., Francis, J., Islam, R. et al. (2017). A guide to using the Theoretical Domains Framework of behaviour change to investigate implementation problems. *Implementation Sci 12*, 77 doi:10.1186/s13012-017-0605-9

Attanayake, V., McKay, R., Joffres, M., Singh, S., Burkle, F Jr, Mills E. (2009). Prevalence of mental disorders among children exposed to war: a systematic review of 7,920 children. *Med Confl Surviv. 25*, 4–19.

Blackmore, R., Gray, K. M., Boyle, J. A., Fazel, M., Ranasinha, S., Fitzgerald, G., Misso, M., & Gibson-Helm, M. (2020) Systematic Review and Meta-analysis: The Prevalence of Mental Illness in Child and Adolescent Refugees and Asylum Seekers. *J Am Acad Child Adolesc Psychiatry, 59(6)*, 705-714. doi: 10.1016/j.jaac.2019.11.011.

Braun, V. & Clarke, V. (2006) Using thematic analysis in psychology, *Qualitative Research in*

Psychology, 3:2, 77-101, DOI: 10.1191/1478088706qp063oa

Brewin, C. (2011). The nature and significance of memory disturbance in posttraumatic stress disorder. *Annual Review of Clinical Psychology, 7*, 203-27.

Child Outcomes Research Consortium. *Child Revised Impact of Events Scale* (Farsi). https://www.corc.uk.net/outcome-experience-measures/child-revised-impact-of-events-scale/

Dalgleish, T., & Werner-Seidler, A. (2014). Disruptions in autobiographical memory processing in depression and the emergence of memory therapeutics. *Trends in Cognitive Sciences, 18*, 596-604.

Edwards, T. C., Huebner, C. E., Connell, F. A., and Patrick, D.L. (2002) Adolescent quality of life, part I: conceptual and measurement model. *J Adolesc 25*:275-286.

Ehring T, et al. (2011). The Perseverative Thinking Questionnaire. *J Behav Ther Exp Psychiatry. 42*, 225–232

Elrha (2015). *The humanitarian health evidence review*.

Frontiers in Psychiatry (2019). Strengthening child and adolescent mental health services and systems in lower-and-middle-income countries (LMICs). (https://www.frontiersin.org/research-topics/8705/strengthening-child-and-adolescent-mental-health-camh-services-and-systems-in-lower-and-middle-incom#overview

HAMI (2013). Afghan refugees/returnees: Challenges & opportunities

Hitchcock, C., Nixon, R. D., Weber, N. (2014). A review of overgeneral memory in child psychopathology. *British Journal of Clinical Psychology, 53*, 170-93

Jalali-Farahani et al. (2019). Health-related quality of life in Iranian Adolescents. *BMC, 17.*

Juengsiragulwit, D. (2015). Opportunities and obstacles in child and adolescent mental health services in low- and middle-income countries: a review of the literature. WHO South-East Asia Journal of Public Health | July–December 2015 | 4 (2)

Kalantari, M., Yule, W., Dyregrov, A., Neshat-Doost, H., Ahmadi, S. J. (2012). Efficacy of writing for recovery on traumatic grief symptoms of Afghani refugee bereaved adolescents: A randomized control trial. *OMEGA - Journal of Death and Dying*, *65*(2):139-150. doi:10.2190/OM.65.2.d

Kleim, B., Graham, B., Fihosy, S., Stott, R., & Ehlers, A. (2014). Reduced specificity in episodic future thinking in posttraumatic stress disorder. *Clinical Psychological Science, 2,* 165-173. 10.1177/2167702613495199.

Kohrt, B. A., Asher, L., Bhardwaj, A., Fazel, M., Jordans, M., Mutamba, B. B., … Patel, V. (2018). The Role of Communities in Mental Health Care in Low- and Middle-Income Countries: A

Meta-Review of Components and Competencies. *International Journal of Environmental Research and Public Health, 15(6)*, 1279. doi:10.3390/ijerph15061279

Kraemer H, et al., (2002). Mediators and moderators of treatment effects in randomized clinical trials. *Arch Gen Psychiatry, 59*: 877-883

Lustig, S. L., Kia-Keating, M., Knight, W. G., Geltman, P., Ellis. H., et al. (2004) Review of child and adolescent refugee mental health. *J Am Acad Child Adolesc Psychiatry, 43(1)*, 24–36

Lyubomirsky, S.et al. (1995). Effects of self-focused rumination on negative thinking and interpersonal problem solving. *J Pers Soc Psychol., 69*, 176-190

Marks, D.F., & Yardley, L. (2004). *Research methods for clinical and health psychology*. London: Sage.

Martins, K., Barry, T. J., Takano, K., & Raes, F. (2019). The transportability of memory Specificity Training (MeST); A dapting an intervention derived from experimental psychology to ne clinical practices. *BMC Psychology, 7*, 5. Doi: 10.1186/s40359-019-0279-y

MacKinnon, D. P., Fairchild, A. J., & Fritz, M. S. (2007). Mediation analysis. *Annual Review of Psychology, 58*, 593–614. https://doi.org/10.1146/annurev.psych.58.110405.085542

Miller, K. E., Omidian, P, Quraishy, A. S, Quraishy, N., Nasiry, M. N., Nasiry, S., Karyar, N. M., & Yaqubi, A. A. (2006). The Afghan symptom checklist: a culturally grounded approach to mental health assessment in a conflict zone. *Am J Orthopsychiatry, 76*(4), 423-433. doi: 10.1037/0002-9432.76.4.423.

Mirabolfathi, V., Schweizer, S., Moradi, A., & Jobson, L. (in press). Affective working memory capacity in refugee adolescents. Psychological Trauma: Theory, Research, Practice and Policy.

Mohammadi, M. R., Arman, S., Khoshhal Dastjerdi, J., Salmanian, M., Ahmadi, N., Ghanizadeh, A., … Motavallian, A. (2013). Psychological problems in Iranian adolescents: application of the self-report form of strengths and difficulties questionnaire. *Iranian Journal of Psychiatry, 8(4),* 152–159.

Moradi, A. R., Moshirpanahi, S., Parhon, H., Mirzaei, J., Dalgleish, T., & Jobson, L. (2014). A pilot randomized controlled trial investigating the efficacy of memory specificity training in improving symptoms of posttraumatic stress disorder. *Behaviour Research and Therapy, 56*, 68–74. doi: 10.1016/j.brat.2014.03.002

Morina, N., von Lersner, U., & Prigerson, H. G. (2011). War and bereavement: Consequences for mental and physical distress. *PLoS One, 6*, e22140.

Mundt, J. C., Marks, I. M., Shear, K., & Greist, J. H. (2002). The work and social adjustment scale:

A simple measure of impairment in functioning. *British Journal of Psychiatry, 180,* 461-464.

Neshat-Doost, H. T., Dalgleish, T., Yule, W., Kalantari, M., Ahmadi, S. J., Dyregrov, A., & Jobson, L. (2013). Enhancing autobiographical memory specificity through cognitive training: An intervention for depression translated from basic science. *Clinical Psychological Science, 1,* 84-92. Doi: 10.1177/2167702612454613

Neshat Doost, H. T., Nouri, N., Molavi, H., Kalantari, M., Mehrabi, H. (2006). Standardization of Mood and Feeling Questionnaire. *Journal of Psychology, 9*, 334-351.

Neshat-Doost, H. Yule, W., Kalantari, M., Rezvani, S., Dyregrov, A. & Jobson, L. (2013). Reduced autobiographical memory specificity in bereaved Afghan adolescents. *Memory, 22.* 10.1080/09658211.2013.817590.

Panter-Brick, C., Eggerman, M., Gonzalez, V., & Safdar, S. (2009). Violence, suffering, and mental health in Afghanistan: a school-based survey. *Lancet (London, England)*, *374*(9692), 807–816. https://doi.org/10.1016/S0140-6736(09)61080-

Panter-Brick, C., Goodman, A., Tol, W., & Eggerman, M. (2011). Mental health and childhood adversities: a longitudinal study in Kabul, Afghanistan. *Journal of the American Academy of Child and Adolescent Psychiatry*, *50*(4), 349–363. https://doi.org/10.1016/j.jaac.2010.12.001

Pedneault, J. (7 October 2019). Afghan's silent mental health crisis. *Human Rights Watch.*

Raes, F., Williams, J. M. G., & Hermans, D. (2009). Reducing cognitive vulnerability to depression: A preliminary investigation of MEmory Specificity Training (MEST) in inpatients with depressive symptomatology. *Journal of Behavioural Therapy and Experimental Psychiatry, 40,* 24-38. DOI: 10.1016/j.jbtep.2008.03.001

Reynolds, C.R. and Richmond, B.O. (1978) "What I think and Feel: A Revised Measure of Children's Manifest Anxiety", Journal of Abnormal Psychology, vol. 6(2), pp. 271-280.

Rasmussen A, Ventevogel P, Sancilio A et al. (2014). Comparing the validity of the self-reporting questionnaire and the Afghan symptom checklist: dysphoria, aggression, and gender in transcultural assessment of mental health. *BMC Psychiatry; 14*: 206. https://doi.org/10.1186/1471-244X-14-20648

Sexton, K. & Dugas, M. J. (2008). Cognitive avoidance questionnaire, *J Anxiety Disord, 22*, 355-70.

Sloan, D.M. et al., (2018). A brief exposure-based treatment vs CPT for PTSD: A randomized non-inferiority clinical trial. *JAMA Psychiatry, 75*, 233-39.

Sutherland, K., & Bryant, R. A. (2008). Social problem solving and autobiographical

memory in posttraumatic stress disorder. *Behaviour Research and Therapy, 46,* 154-161.

Taghavi, M.R. & Alishahi, M., J. (2004). Reliability and validity of the revised children's manifest anxiety scale. *Journal of Psychology, 28*, 342-357

Taheri, E. et al. (2016). Cognitive therapy versus behavioural activation therapy in the treatment of social anxiety disorder. *Journal of Fundamentals of Mental Health, 18*, 294

The Economist, March 2019. What disasters reveal about mental-health care. (https://www.economist.com/international/2019/03/16/what-disasters-reveal-about-mental-health-care)

Tyrer RA, Fazel M (2014) School and community-based interventions for refugee and asylum seeking children: A systematic review. *PLoS ONE 9(2)*, e89359.Doi:10.1371/journal.pone.0089359

UN Children's Fund (2019). *UNICEF Afghanistan Humanitarian Situation Report*

United Nations Office for the Coordination of Humanitarian Affairs (2017). *2018 Afghanistan Humanitarian Needs Overview.*

UNICEF (2019a). *Afghanistan: Adolescent health and development.* https://www.unicef.org/afghanistan/adolescent-health-and-development

UNICEF (2019b) *Adolescents overview*. https://data.unicef.org/topic/adolescents/overview/

United Nations High Commissioner for Refugees (2019). *Afghanistan*. https://www.unhcr.org/en-au/afghanistan.html

United Nations High Commissioner for Refugees (2020). Operation Portal Refugee Situations: Afghanistan

Werner-Seidler, A, et al., Raes, F., Jobson, L., ..(2018). A cluster RCT comparing group MEST to group psychoeducation and supportive counselling in the treatment of recurrent depression. *BRAT, 105*, 1-9.

Williams, J. M. G., Barnhofer, T., Crane, C., Hermans, D., Raes, F., Watkins, E., & Dalgleish, T. (2007). Autobiographical memory specificity and emotional disorder. *Psychological Bulletin, 133*,122–148.doi: 10.1037/0033-2909.133.1.122

World Health Organization (2019). *Adolescent Health and Development.* (http://www.searo.who.int/entity/child_adolescent/topics/adolescent_health/en/)

Yazdi, Z., Sadeghniiat-Haghighi, K., Zohal, M. A., & Elmizadeh, K. (2012). Validity and reliability of the Iranian version of the insomnia severity index. *The Malaysian journal of medical sciences: MJMS, 19(4),* 31–36.

Zia, M. S., Afkhami, E., Tavakoli, M., Neshat-Doost, H., & Jobson, L. (in press). A Brief Clinical Report Documenting a Novel Therapeutic Technique (Memory Specificity Training) for

Depression: A summary of two preliminary randomized controlled trials. *Behaviour and Cognitive Psychotherapy*

Randomized Clinical Trial to evaluate efficacy, feasibility and appropriateness of MEmory Training for Recovery- Adolescent (METRA) among war-affected adolescents: A structured summary of a study protocol for a randomized controlled trial – Version 2

Abstract

Background: Adolescent refugees and war-affected youth in low- and middle-income countries (LMICs) generally do not receive evidence-based psychological interventions, as most interventions are complex, require specialist knowledge, and are prohibitively expensive. This study aims to investigate the efficacy of MEmory Training for Recovery-Adolescent (METRA) in improving psychological symptoms (posttraumatic stress disorder, depression) in war-affected adolescents delivered in LMIC humanitarian contexts. Our secondary aims are to examine the feasibility and appropriateness of METRA, ~~explore the mechanisms mediating treatment effects, and to include a cost-analysis of METRA in LMIC humanitarian contexts~~.(CHANGE 1: Removed mechanisms study and cost-analysis. These aspects were not included due to security reasons – assessment sessions had to be as short as possible for safety of youth and researchers)

Methods: We will use mixed methods to assess feasibility, appropriateness, and efficacy. ~~The project will also include an embedded mechanism study and a cost-analysis component.~~

Discussion: Research outputs include an evaluation of METRA, an innovative, low-intensity, freely-available intervention that can be delivered by individuals with minimal training in humanitarian contexts. We anticipate that refugee and war-affected adolescents who experience high levels of psychological distress will benefit from this research.

Trial registration: We have submitted our trial for registration on the Australian New Zealand Clinical Trials Registry.

Keywords

Adolescent; Refugee; War-affected; Memory Training for Recovery; Trauma; Depression

Administrative information

The trial is funded by R2HC 2019 ELRHA funding (44708).

Title {1}	Randomized Clinical Trial to evaluate efficacy, feasibility and appropriateness of MEmory Training for Recovery- Adolescent (METRA) among war-affected adolescents: a structured summary of a study protocol for a randomized controlled trial
Trial registration {2a and 2b}.	Australian New Zealand Clinical Trials Registry (Trial Id: ACTRN12621001160820)
Protocol version {3}	September 2021. Version 2.
Funding {4}	The trial is funded by a Research for Health in Humanitarian Crises Grant (ELRHA) (44708). Funders: UKAID, Wellcome Trust, National Institute for Health Research
Author details {5a}	1) Associate Professor Laura Jobson – School of Psychological Sciences and Turner Institute for Brain and Mental Health, Monash University, Melbourne Australia<br>2) Dr Sayed Jafar Ahmadi - Shaheed Prof. Rabbani Education University, Kabul, Afghanistan<br>3) Zainab Mosavi – Behrawan Research and Psychological Services Organization, Kabul, Afghanistan<br>4) Sayed Rohollah Rezvani – Department of Psychology, University of Isfahan, Isfahan, Iran<br>5) Associate Professor Arul Earnest – Biostatistics Unit, School of Public Health and Preventive Medicine, Monash University, Melbourne, Australia<br>6) Dr Daniel McAvoy – Centre for Humanitarian Leadership, Deakin University, Melbourne Australia

Name and contact information for the trial sponsor {5b}	Monash University
Role of sponsor {5c}	N/A

Introduction

Background and rationale {6a}

War-affected and refugee adolescents in low- and middle-income countries (LMICs) experience concerning levels of psychological distress, including posttraumatic stress disorder (PTSD), depression, and anxiety (Attanayake, McKay, Joffres, Singh, Burkle, & Mills, 2009; Blackmore et al., 2020; Morina, von Lersner, & Prigerson, 2011; Tyrer & Fazel, 2014). Afghanistan has endured a long-history of armed conflict, poverty, and social injustice, which has significantly impacted on the mental health of Afghan youth (Panter-Brick, Eggerman, Gonzalez, & Safdar, 2009; Panter-Brick, Goodman, Tol, & Eggerman, 2011). Research has shown high levels of distress and poor mental health among Afghan youth, including PTSD, depression, anxiety and somatoform symptoms (Alemi et al., 2018). Indeed, in a recent study we found that all Afghan adolescent refugees recruited for our research were experiencing concerning levels of PTSD and depression (Mirabolfathi, Schweizer, Moradi, & Jobson, 2019). These symptoms severely affect an adolescent's life, including long-lasting cognitive, emotional, social and academic/vocational problems (Neshat-Doost, Yule, Kalantari, Rezvani, Dyregrov, & Jobson, 2013). Addressing psychological concerns in the critical developmental period of adolescence is imperative (Neshat-Doost et al., 2013; The Economist, 2019). However, very few refugee and war-affected adolescents in LMICs receive evidence-based interventions, due to high costs, limited mental health services and a shortage of skilled professionals (Juengsiragulwit, 2015; Zia, Afkhami, Tavakoli, Neshat-Doost, & Jobson, in press; Kohrt et al., 2018). The needs of adolescents in LMICs have not received sufficient attention in psychology and psychiatry research (Frontiers in Psychiatry, 2019). In order to meet these identified needs, we propose that MEmory Training for Recovery-Adolescent (METRA), a low-intensity, accessible and scalable evidence-based intervention, will improve adolescent mental health (specifically depression and PTSD), .

Trauma and Memory

Those suffering from depression and PTSD, including adolescent refugees and war-affected youth (Neshat-Doost et al., 2013), exhibit certain disruptions in their autobiographical remembering (i.e., remembering personal experiences from one's life) (Brewin, 2011; Dalgleish & Werner-Seidler, 2014). Trauma memories are often intrusive and distressing (Brewin, 2011) and PTSD sufferers have considerable difficulties remembering specific events from their lives (e.g., "I attended Adina's party on Friday"). Instead, they provide overgeneral memories (OGM) of events ("I was attending a party every weekend") (Williams et al., 2007). OGM develops as a cognitive avoidance strategy in response to the distressing memories one may experience (Williams et al., 2007).

OGM is problematic as it is associated with impaired social problem-solving, cognitive avoidance, rumination, impairments in executive control, difficulty imagining specific events in the future (Kleim, Graham, Fihosy, Stott, & Ehlers, 2014; Sutherland & Bryant, 2008; Williams et al., 2007), and difficulty accessing specific information about the past, which interferes with the ability to update distressing memories (Moradi, Moshirpanahi, Parhon, Mirzaei, Dalgleish, & Jobson, 2014; Williams et al., 2007). Each of these processes are integral to recovery from PTSD and depression (Moradi et al., 2014; Neshat-Doost et al., 2013). Not surprisingly, an OGM retrieval style in adolescents has been found to predict on-going psychological difficulties, which can persist well into adulthood affecting social functioning and academic/vocational attainment (Hitchcock, Nixon, & Weber, 2014).

There is enormous untapped potential to improve the psychological functioning of refugee and war-affected adolescents by targeting these memory difficulties that underpin posttraumatic psychological distress. In response to this, we propose that MEmory Training for Recovery-Adolescents (METRA), a novel intervention for use in humanitarian contexts (Moradi et al., 2014; Neshat-Doost et al., 2013), may have potential in improving the psychological adjustment of adolescents.

Feasibility and Piloting of METRA Modules

METRA is an evidence-based, low-intensity, easily disseminable, transdiagnostic training package. It is comprised of two modules that target major cognitive features underpinning posttrauma psychological difficulties experienced by adolescent refugees; OGM and trauma/distressing memories.

Module 1 is a memory specificity training that teaches adolescents to recall specific positive, negative and neutral everyday memories from their lives (targets OGM) (Raes, Williams, & Hermans, 2009). Module 2 is a writing for recovery, written exposure training (e.g., Kalantari, Yule, Dyregrov, Neshat-Doost, & Ahmadi, 2012; Sloan et al., 2018; Sloan & Marx, 2019) targeting distressing and trauma memories. Adolescents are supported in writing about their past including thoughts and feelings. The results of our pilot research, investigating the efficacy of the components of METRA, have been promising in the treatment of depression and PTSD in refugee adolescents and war veterans in LMICs (see Ahmadi, Kajbaf, Neshat Doost, Dalgleish, Jobson, & Mosavi, 2018; Kalantari et al., 2012; Moradi et al., 2014; Neshat-Doost et al., 2013). Furthermore, our studies have shown preliminary support that these modules were feasible in humanitarian settings, the training was well received by participants and organizations, and adolescents reported satisfaction and were motivated (Ahmadi et al., 2018; Kalantari et al., 2012; Moradi et al., 2014; Neshat-Doost et al., 2013).

METRA aligns with humanitarian initiatives aiming to deliver more accessible, low-resource (i.e., group-based, unskilled facilitator) psychosocial evidence-based interventions (Elrha, 2015). METRA, a low-intensity intervention, seems feasible in humanitarian settings and has potential for reducing PTSD and depression symptoms in refugee adolescents. There is a need to better understand the mechanisms driving the therapeutic effects of METRA and the costs associated with implementing METRA in humanitarian contexts. This project addresses identified humanitarian mental health research gaps (Elrha, 2015) by investigating the efficacy and feasibility of scaling-up a low-intensity modular transdiagnostic psychosocial interventions for adolescents. It also includes qualitative methods to examine appropriateness and acceptability of METRA ~~and a health-economic component to undertake cost-analysis of the intervention~~.

Objectives {7}

Primary Objectives

1) Investigate the efficacy of METRA in improving psychological symptoms (posttraumatic stress disorder, depression) in adolescents living in refugee-like and humanitarian situations.

Secondary Objectives

2) Investigate the feasibility and appropriateness of METRA for adolescents living in refugee-like and humanitarian situations delivered in LMIC humanitarian contexts.

3) ~~Examine the mechanisms mediating treatment effects.~~

~~4) Undertake a cost-analysis of METRA in LMIC humanitarian contexts~~.

Trial design {8}

This is a randomised controlled trial (RCT) comparing METRA to treatment as usual (TAU), with an embedded qualitative component. Participants will be assessed; 1) at baseline, 2) at post-Module 1, 3) at post-Module 2, and 4) at ~~6-month~~ 3-month follow-up (CHANGE 2: Follow-up was changed to 3-months. This decision was made due to security issues and increased movement within Afghanistan following the change in government). The first three assessments are face-to-face assessment and will include all measures. The follow-up assessments will be conducted by phone/skype/zoom and will include the primary and secondary outcomes. Assessments will be conducted by independent raters who have no therapeutic relationship with participants and are blind to condition.

Methods: Participants, interventions and outcomes

Study setting {9}

The Afghan humanitarian crisis has been identified as one of the world's most complex, severe and protracted humanitarian emergencies, with no sign of abatement (UNHCR, 2017). Afghanistan has one of the world's youngest populations, with adolescents accounting for around 26 per cent of the population (UNICEF, 2019a). The developmental process of growing up and being an adolescent in Afghanistan is very difficult (UNICEF, 2019a). Adolescents in Afghanistan are affected everyday by conflict and violence, on-going insecurity, and periodic natural disasters, which can impede development (UNICEF, 2019a). Compounding this, Afghan adolescents have poor access to healthcare, education, nutrition and have few livelihood opportunities, which can have long-term impacts on their mental and physical health (UNICEF, 2019a). Unsurprisingly, then, many Afghan adolescents are experiencing concerning levels of psychological distress (Ashrafi, 2019; Pedneault, 2019; UNOCHA, 2017; UNHCR, 2019).

There are around 2.6 million displaced Afghans, half of whom are under the age of 18 (UNHCR, 2019). Since 2020 many Afghan refugees are returning to Afghanistan, with many returnees and internally-displaced peoples residing in Herat (third largest city in Afghanistan situated near the border of Iran) and Kabul (Afghan capital) (AFP, 2020; Amnesty, 2021; UNHCR, 2020). The focus on sustainable return and reintegration of Afghan refugees, alongside the COVID-19 pandemic, has meant that many Afghan adolescents are returning to Afghanistan, reducing their access to essential

mental health services (AFP, 2020; HAMI, 2013; UNOCHA, 2017). The conflict in Afghanistan has resulted in the closure and destruction of health facilities. Conflict and insecurity continue to impact negatively on adolescent returnee's health; it is estimated that around 2 million children/adolescents will require humanitarian health assistance but face limited access to services (UNOCHA, 2017). Thus, Afghanistan context has been categorised as complex, very high crisis and impact, and with extreme humanitarian access constraints (ACAPS, 2019; HAMI, 2013).

This study will take place in one ~~two~~ setting within Afghanistan – Kabul ~~and Herat~~ (CHANGE 3: The study only occurred in Kabul. It was deemed too unsafe to conduct the study in Herat). Both of these settings have been identified as a high-risk complex humanitarian crisis (ACAPS, 2019; United Nations Office for the Coordination of Humanitarian Affairs, 2017, 2018; UN Children's Fund, 2019). Ensuring the mental health of Afghan adolescents is essential for the stability, security and prosperity of Afghanistan (UNHCR, 2018).

Eligibility criteria {10}

Afghan refugee/returnee/war-affected adolescent girls aged 10-19 years with elevated psychological distress. This age range aligns with our pilot work and definitions of 'adolescent' (UNICEF, 2019b; WHO, 2019). As in our pilot studies, elevated psychological distress is defined as >30 on the Persian Child Revised Impact of Event Scale-13 (Child Outcomes Research Consortium, 2019) and/or >12 on the Persian Mood and Feeling Questionnaire – Short form (Neshat-Doost et al., 2006). Participants will be recruited in Kabul. Exclusion Criteria: a) high levels of suicidality, b) unmanaged psychosis/manic episodes in past month, and c) presence of head trauma/organic brain damage.

Who will take informed consent? {26a}

Researchers will gain informed consent from the adolescents and their guardians.

Additional consent provisions for collection and use of participant data and biological specimens {26b}

N/A

Interventions

Explanation for the choice of comparators {6b}

Treatment as usual (TAU): Local NGOs will provide the course of intervention that they deem appropriate. No specific instructions will be given as to what TAU should entail, except not including elements specific to METRA. TAU will be documented ensuring understanding of the duration, frequency and type of treatment administered.

Intervention description {11a}

METRA: Module 1: Memory specificity training (Ahmadi et al., 2018; Moradi et al., 2014; Neshat-Doost et al., 2013; Raes et al., 2009; Martens et al., 2019) is a manualized training delivered over five 60-minute sessions to groups of 6-8 adolescents. MEST aims to enhance memory specificity through practice. Session 1 provides psycho-education about mental health and memory. Participants practice recalling memories in response to positive and neutral cues, with support from the group facilitator. Attention is paid to the contextual, spatio-temporal and sensory-perceptual details of the memories (Raes et al., 2009). Participants' responses are discussed in the group. At the end of the session, homework exercises are introduced; for 10 cues (positive and neutral) participants need to generate a specific memory and are instructed to write down a 'specific memory of the day' every evening of the coming week (Raes et al., 2009). Session 2 starts with a brief summary of Session 1, the homework exercises are discussed and the Session then follows the same format as Session 1, with further practice focusing on recalling memories in response to positive and neutral cues. At the end of Session 2, the homework is explained; participants need to generate two different specific memories for 10 cues (positive and neutral) and write down two different 'specific memories of the day' every evening of the coming week (Raes et al., 2009). Session 3 is very similar to Session 2. However, in Session 3, participants also need to work with negative cues. The homework exercises are similar to those outlined in Session 2, but now also include negative cues. Session 4 involves further exercises using negative and ('counterpart') positive cues. It is also explained how overgeneral thinking can be addressed by recalling a single specific experience and examples are discussed to promote metacognitive awareness of when participants are starting to shift to unspecific thinking or more general retrieval (Raes et al., 2009). Session 5 includes further practice and a summary of Module 1. Module 1 focuses on everyday remembering.

Module 2: Writing for Recovery is a written exposure training involves five sessions (Kalantari et al., 2012; Sloan et al., 2018). In the first session the purpose of Module 2 is outlined. In the following sessions, the facilitator simply reads the instructions and the participant completes the writing task; writing about their trauma including thoughts and feelings. After 30 minutes, the facilitator instructs the participants to stop writing (CHANGE 4: Timing of sessions reduced due to

security concerns).

Criteria for discontinuing or modifying allocated interventions {11b}

Discontinue trial if participants report significant distress or a significant proportion of participants report significant increase in symptomatology. This will be based on the reports and observations of the facilitators and decisions to continue/discontinue will be made by the trial monitoring body.

Strategies to improve adherence to interventions {11c}

Group facilitators will be trained and receive supervision. A random 25% of the audio-recorded treatment sessions will be rated for manual adherence. To reduce group contamination, participants will be requested to not discuss treatment with others.

Relevant concomitant care permitted or prohibited during the trial {11d}

N/A

Provisions for post-trial care {30}

Referrals to local mental health services and NGOs will be made for participants requiring further psychological care.

Outcomes {12}

Primary Outcomes: self-reported symptoms of PTSD (Child Outcomes Research Consortium, 2019) and depression (Neshat-Doost et al., 2006). We will assess statistical significance of change in the levels of PTSD and depression symptoms in the intervention and control groups.

Secondary Outcomes: Afghan-specific symptoms (Miller et al., 2006; Rasmussen et al., 2014); anxiety (Reynolds & Richmond, 1978); and internalising/externalising problems (Alavi et al., 2009).

Qualitative data: Interviews with adolescents in the METRA arm of the trial will be conducted to examine appropriateness and feasibility of METRA.

The measures and interviews will be conducted in Pashto and Dari.

Participant timeline {13}

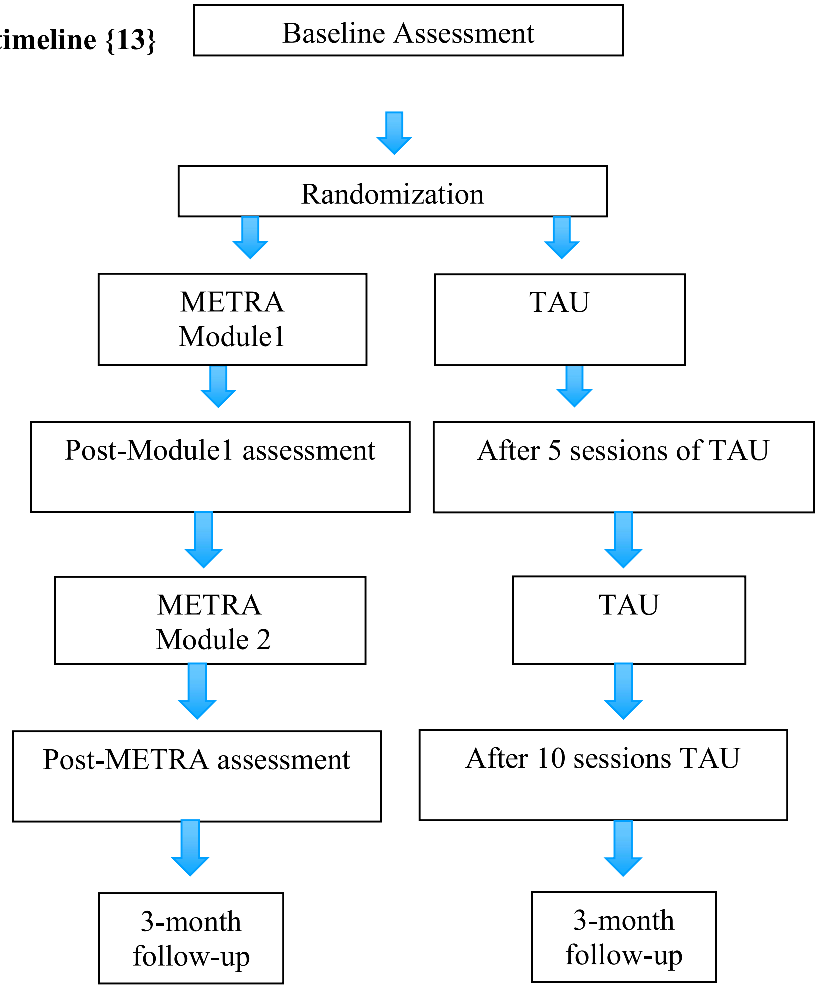

Figure 1: Flowchart of participant recruitment and assessment

Sample size {14}

A priori power analysis was undertaken using G*Power for depression and PTSD outcomes to detect a small to moderate interaction effect (f=0.15)[18,33] between group (intervention, control) and time (pre-test, post-test) at an alpha-level of 0.05. A sample size of 90 participants would achieve power >0.80. The sample size estimate was re-adjusted to allow for attrition and we aim to recruit >100 participants.

Recruitment {15}

Participants will be recruited through local non-government agencies and schools in Herat and Kabul.

Assignment of interventions: allocation

Sequence generation {16a}

After eligibility is confirmed and baseline data collected, participants will be randomly assigned in a 2:1 ratio to METRA or TAU (CHANGE 5: Change in random assignment ratio approach due to resource and ethical reasons). We have adopted an unequal randomization approach for resource and ethical reasons to maximize the number of participants in the treatment arm. This decision was made because following the change of government, there was increased insecurity and most international agencies withdrew from Afghanistan. Thus, there are now very few youth health interventions available that can be considered TAU. This unequal randomization approach has been employed in previous trauma-focused RCTs (including those conducted in LMICS) (Bass et al., 2016; Rae Olmsted et al., 2020) and the decision should not impact power for our statistical tests nor changed the scientific rigor of the study (Torgerson & Torgerson, 2008).

Concealment mechanism {16b}

We will use consecutively numbered sealed opaque envelopes to conceal the allocation.

Implementation {16c}

The research team will oversee generation of the allocation sequence, participant enrollment, and assigning participants to interventions.

Assignment of interventions: Blinding

Who will be blinded {17a}

Outcome assessors will be blind to hypotheses and group allocation. Group facilitators will be blind to hypotheses.

Procedure for unblinding if needed {17b}

Not applicable

Data collection and management

Plans for assessment and collection of outcomes {18a}

Data will be collected at; 1) baseline, 2) post-Module 1, 3) post-Module 2, and 4) ~~6-month~~ 3-month follow-up. Assessments will be conducted by independent raters who have no therapeutic relationship with participants and are blind to condition. All assessors will be trained and supervised in assessment procedures. All questionnaires have good reliability and validity and have been previously used cross-culturally.

Plans to promote participant retention and complete follow-up {18b}

Participants will be contacted using a variety of media, including phone, email and social media to promote retention and follow-up completion.

Data management {19}

Data will be coded, entered and stored in Kabul ~~and Herat~~ on password protected computer systems. Anonymized data will be shared amongst the research team.

Confidentiality {27}

Personal information about potential and enrolled participants will only be collected, shared, maintained and monitored by the research teams at each site. De-identified data will be shared between the team.

Plans for collection, laboratory evaluation and storage of biological specimens for genetic or molecular analysis in this trial/future use {33}

N/A

Statistical methods

Statistical methods for primary and secondary outcomes {20a}

All primary analyses will be on intent-to-treat principle, with all randomized participants analyzed in their allocation condition.

Objective 1 will be examined using linear mixed effects models with intervention type, time, and intervention by time interaction as fixed factors (CHANGE 6: This was changed to generalized estimating equations following reviewer feedback. However, the results were the similar when using linear mixed effects models). Repeated assessments of individuals will be modelled as random intercept. Of primary interest will be the intervention by time interaction, which will compare the levels of change over time in outcomes of the METRA and TAU groups. Levels of significance will be set at $\alpha<.05$ and hypothesis tests will be 2-sided. Objective 2: Thematic analysis of qualitative data (Atkins et al., 2017; Braun & Clark, 2006; Marks & Yardley, 2004) will assess feasibility and appropriateness.

Interim analyses {21b}

Not applicable

Methods for additional analyses (e.g. subgroup analyses) {20b}

Exploratory Analyses: Potential moderators (age, gender, trauma exposure, location, refugee experience) of intervention response will be explored with linear mixed models.

Methods in analysis to handle protocol non-adherence and any statistical methods to handle

missing data {20c}

N/A

Plans to give access to the full protocol, participant level-data and statistical code {31c}

N/A

Oversight and monitoring

Composition of the coordinating centre and trial steering committee {5d}

The Trial Steering Committee will provide overall supervision of the project. It will include independent members who will monitor trial progress, functioning of the partnership and ensure local and community involvement.

Composition of the data monitoring committee, its role and reporting structure {21a}

Not applicable

Adverse event reporting and harms {22}

Should an adverse effect be reported, the Chief Investigators will be notified immediately and a plan to resolve the serious adverse effect will be formulated. Depending on the nature of the adverse event, the Chief Investigators and research team will determine the appropriate course of action and then the research team will discuss the situation fully with the participant. A complete list of adverse effects, the steps taken to resolve them, and the results of those steps, will be reported to the Ethics Review Committee, Trial Steering Committee, Kabul University and Behrawan monthly, while serious adverse effects will be reported within one business day.

Frequency and plans for auditing trial conduct {23}

The Trial Steering Committee will provide overall supervision of the project.

Plans for communicating important protocol amendments to relevant parties (e.g. trial participants, ethical committees) {25}

We will report changes to the ethic committees and trial registry.

Dissemination plans {31a}

The results will be published in peer-reviewed journal articles. There will also be an Engagement and Communication Strategy that will ensure key local (Afghanistan) and international stakeholders are informed about the findings.

Discussion

The complex humanitarian context of Herat and Kabul present operational challenges, as several of the universities are in the process of re-establishing themselves. However, our collaborations are well-established and we will follow the research protocols that were employed in our pilot work in this region.

Trial status

Protocol version number 2; September 2021.

Abbreviations

PTSD: Posttraumatic stress disorder

METRA: MEmory Training for Recovery – Adolescents

Declarations

Acknowledgements : N/A

Authors' contributions {31b}

LJ, S.A., A.R.M., H.N-D, V.M. are the Chief Investigators; they conceived the study, led the proposal and protocol development. FA, ZM, SR and DM contributed to study design and to development of the proposal. KV is the lead trial health economist and LB and AE are the lead trial biostatisticians. All authors read and approved the final manuscript.

Funding {4}

The trial is funded by R2HC 2019 ELRHA funding (44708).

Availability of data and materials {29}

The research team will have the final dataset and this dataset will be available by contacting the researchers.

Ethics approval and consent to participate {24}

We will obtain ethical approval for the study from the Ministry of Health, Afghanistan.

Consent for publication {32}

Not applicable.

Competing interests {28}

The principal investigators have no competing interests.

References

Abela, J. R. Z., Vanderbilt, E., & Rochon, A. (2004). A test of the integration of the response styles and social support theories of depression in third and seventh grade children. *Journal of Social and Clinical Psychology, 23(5),* 653-674. doi: 10.1521/jscp.23.5.653.50752

ACAPS (2019). Crisis updates: Afghanistan. (https://www.acaps.org/country/afghanistan/crisis/complex-crisis)

AFP (2020) *Coronavirus: Nearly 70 000 Afghans return home after fleeing virus hit Iran*.

Ahmadi, S. J., Kajbaf, M. B., Neshat Doost, H. T., Dalgleish, T., Jobson, L., Mosavi, Z. (2018). The efficacy of memory specificity training in improving symptoms of post-traumatic stress disorder in bereaved Afghan adolescents. *Intervention, 16*, 243-248.

Alavi, Md & Mohammadi, Mohammad-Reza & Md, J & Tehranidoost, M & Shahrivar, Zahra & Saadat, Soheil. (2009). The Farsi version of the strength and difficulties questionnaire self-report form: The normative data and scale properties. *Iranian Journal of Child Neurology. 3*.

Alemi, Q., Stempel, C., Koga, P.M. *et al.* Risk and protective factors associated with the mental health of young adults in Kabul, Afghanistan. *BMC Psychiatry 18,* 71 (2018). https://doi.org/10.1186/s12888-018-1648-4

Amnesty (2021, March 30). Afghanistan: Country's four million internally displaced need urgent support amid pandemic. https://www.amnesty.org/en/latest/news/2021/03/afghanistan-countrys-four-million-internally-displaced-need-urgent-support-amid-pandemic/

Afghanistan: Country's four million internally displaced need urgent support amid pandemic

Ashrafi, F. (2019). Fatemeh Ashrafi, the director of HAMI "Iran's immigrant politics is based on the Islamic brotherhood and understanding". http://hamiorg.org/en/?p=3609

Atkins, L., Francis, J., Islam, R. et al. (2017). A guide to using the Theoretical Domains Framework of behaviour change to investigate implementation problems. *Implementation Sci 12*, 77 doi:10.1186/s13012-017-0605-9

Attanayake, V., McKay, R., Joffres, M., Singh, S., Burkle, F Jr, Mills E. (2009). Prevalence of mental disorders among children exposed to war: a systematic review of 7,920 children. *Med Confl Surviv. 25*, 4–19.

Bass J, Murray SM, Mohammed TA et al. Randomized Controlled Trial of a Trauma Informed Support, Skills, and Psychoeducation Intervention for Survivors of Torture and Related Trauma in Kurdistan, Northern Iraq. *Glob Health Sci Pract.* 2016 Sep 29;4(3):452-66. doi: 10.9745/GHSP-D-16-00017. PMID: 27609624; PMCID: PMC5042700.

Blackmore, R., Gray, K. M., Boyle, J. A., Fazel, M., Ranasinha, S., Fitzgerald, G., Misso, M., &

Gibson-Helm, M. (2020) Systematic Review and Meta-analysis: The Prevalence of Mental Illness in Child and Adolescent Refugees and Asylum Seekers. *J Am Acad Child Adolesc Psychiatry, 59(6)*, 705-714. doi: 10.1016/j.jaac.2019.11.011.

Braun, V. & Clarke, V. (2006) Using thematic analysis in psychology, *Qualitative Research in Psychology, 3*:2, 77-101, DOI: 10.1191/1478088706qp063oa

Brewin, C. (2011). The nature and significance of memory disturbance in posttraumatic stress disorder. *Annual Review of Clinical Psychology, 7*, 203-27.

Child Outcomes Research Consortium. *Child Revised Impact of Events Scale* (Farsi). https://www.corc.uk.net/outcome-experience-measures/child-revised-impact-of-events-scale/

Dalgleish, T., & Werner-Seidler, A. (2014). Disruptions in autobiographical memory processing in depression and the emergence of memory therapeutics. *Trends in Cognitive Sciences, 18*, 596-604.

Edwards, T. C., Huebner, C. E., Connell, F. A., and Patrick, D.L. (2002) Adolescent quality of life, part I: conceptual and measurement model. *J Adolesc 25*:275-286.

Ehring T, et al. (2011). The Perseverative Thinking Questionnaire. *J Behav Ther Exp Psychiatry. 42*, 225–232

Elrha (2015). *The humanitarian health evidence review*.

Frontiers in Psychiatry (2019). Strengthening child and adolescent mental health services and systems in lower-and-middle-income countries (LMICs). (https://www.frontiersin.org/research-topics/8705/strengthening-child-and-adolescent-mental-health-camh-services-and-systems-in-lower-and-middle-incom#overview

HAMI (2013). Afghan refugees/returnees: Challenges & opportunities

Hitchcock, C., Nixon, R. D., Weber, N. (2014). A review of overgeneral memory in child psychopathology. *British Journal of Clinical Psychology, 53*, 170-93

Jalali-Farahani et al. (2019). Health-related quality of life in Iranian Adolescents. *BMC, 17.*

Juengsiragulwit, D. (2015). Opportunities and obstacles in child and adolescent mental health services in low- and middle-income countries: a review of the literature. WHO South-East Asia Journal of Public Health | July–December 2015 | 4 (2)

Kalantari, M., Yule, W., Dyregrov, A., Neshat-Doost, H., Ahmadi, S. J. (2012). Efficacy of writing for recovery on traumatic grief symptoms of Afghani refugee bereaved adolescents: A randomized control trial. *OMEGA - Journal of Death and Dying*, *65*(2):139-150. doi:10.2190/OM.65.2.d

Kleim, B., Graham, B., Fihosy, S., Stott, R., & Ehlers, A. (2014). Reduced specificity in episodic

future thinking in posttraumatic stress disorder. *Clinical Psychological Science, 2,* 165-173. 10.1177/2167702613495199.

Kohrt, B. A., Asher, L., Bhardwaj, A., Fazel, M., Jordans, M., Mutamba, B. B., … Patel, V. (2018). The Role of Communities in Mental Health Care in Low- and Middle-Income Countries: A Meta-Review of Components and Competencies. *International Journal of Environmental Research and Public Health, 15(6)*, 1279. doi:10.3390/ijerph15061279

Kraemer H, et al., (2002). Mediators and moderators of treatment effects in randomized clinical trials. *Arch Gen Psychiatry, 59*: 877-883

Lustig, S. L., Kia-Keating, M., Knight, W. G., Geltman, P., Ellis. H., et al. (2004) Review of child and adolescent refugee mental health. *J Am Acad Child Adolesc Psychiatry, 43(1)*, 24–36

Lyubomirsky, S.et al. (1995). Effects of self-focused rumination on negative thinking and interpersonal problem solving. *J Pers Soc Psychol., 69*, 176-190

Marks, D.F., & Yardley, L. (2004). *Research methods for clinical and health psychology*. London: Sage.

Martins, K., Barry, T. J., Takano, K., & Raes, F. (2019). The transportability of memory Specificity Training (MeST); Adapting an intervention derived from experimental psychology to ne clinical practices. *BMC Psychology, 7*, 5. Doi: 10.1186/s40359-019-0279-y

MacKinnon, D. P., Fairchild, A. J., & Fritz, M. S. (2007). Mediation analysis. *Annual Review of Psychology, 58*, 593–614. https://doi.org/10.1146/annurev.psych.58.110405.085542

Miller, K. E., Omidian, P, Quraishy, A. S, Quraishy, N., Nasiry, M. N., Nasiry, S., Karyar, N. M., & Yaqubi, A. A. (2006). The Afghan symptom checklist: a culturally grounded approach to mental health assessment in a conflict zone. *Am J Orthopsychiatry, 76*(4), 423-433. doi: 10.1037/0002-9432.76.4.423.

Mirabolfathi, V., Schweizer, S., Moradi, A., & Jobson, L. (in press). Affective working memory capacity in refugee adolescents. Psychological Trauma: Theory, Research, Practice and Policy.

Mohammadi, M. R., Arman, S., Khoshhal Dastjerdi, J., Salmanian, M., Ahmadi, N., Ghanizadeh, A., … Motavallian, A. (2013). Psychological problems in Iranian adolescents: application of the self-report form of strengths and difficulties questionnaire. *Iranian Journal of Psychiatry, 8(4),* 152–159.

Moradi, A. R., Moshirpanahi, S., Parhon, H., Mirzaei, J., Dalgleish, T., & Jobson, L. (2014). A pilot randomized controlled trial investigating the efficacy of memory specificity training in improving symptoms of posttraumatic stress disorder. *Behaviour Research and Therapy, 56*,

68–74. doi: 10.1016/j.brat.2014.03.002

Morina, N., von Lersner, U., & Prigerson, H. G. (2011). War and bereavement: Consequences for mental and physical distress. *PLoS One, 6*, e22140.

Mundt, J. C., Marks, I. M., Shear, K., & Greist, J. H. (2002). The work and social adjustment scale: A simple measure of impairment in functioning. *British Journal of Psychiatry, 180,* 461-464.

Neshat-Doost, H. T., Dalgleish, T., Yule, W., Kalantari, M., Ahmadi, S. J., Dyregrov, A., & Jobson, L. (2013). Enhancing autobiographical memory specificity through cognitive training: An intervention for depression translated from basic science. *Clinical Psychological Science, 1*, 84-92. Doi: 10.1177/2167702612454613

Neshat Doost, H. T., Nouri, N., Molavi, H., Kalantari, M., Mehrabi, H. (2006). Standardization of Mood and Feeling Questionnaire. *Journal of Psychology, 9*, 334-351.

Neshat-Doost, H. Yule, W., Kalantari, M., Rezvani, S., Dyregrov, A. & Jobson, L. (2013). Reduced autobiographical memory specificity in bereaved Afghan adolescents. *Memory, 22.* 10.1080/09658211.2013.817590.

Panter-Brick, C., Eggerman, M., Gonzalez, V., & Safdar, S. (2009). Violence, suffering, and mental health in Afghanistan: a school-based survey. *Lancet (London, England)*, *374*(9692), 807–816. https://doi.org/10.1016/S0140-6736(09)61080-

Panter-Brick, C., Goodman, A., Tol, W., & Eggerman, M. (2011). Mental health and childhood adversities: a longitudinal study in Kabul, Afghanistan. *Journal of the American Academy of Child and Adolescent Psychiatry*, *50*(4), 349–363. https://doi.org/10.1016/j.jaac.2010.12.001

Pedneault, J. (7 October 2019). Afghan's silent mental health crisis. *Human Rights Watch.*

Rae Olmsted KL, Bartoszek M, Mulvaney S, et al. Effect of Stellate Ganglion Block Treatment on Posttraumatic Stress Disorder Symptoms: A Randomized Clinical Trial. *JAMA Psychiatry.* 2020;77(2):130–138. doi:10.1001/jamapsychiatry.2019.3474

Raes, F., Williams, J. M. G., & Hermans, D. (2009). Reducing cognitive vulnerability to depression: A preliminary investigation of MEmory Specificity Training (MEST) in inpatients with depressive symptomatology. *Journal of Behavioural Therapy and Experimental Psychiatry, 40,* 24-38. DOI: 10.1016/j.jbtep.2008.03.001

Reynolds, C.R. and Richmond, B.O. (1978) "What I think and Feel: A Revised Measure of Children's Manifest Anxiety", Journal of Abnormal Psychology, vol. 6(2), pp. 271-280.

Rasmussen A, Ventevogel P, Sancilio A et al. (2014). Comparing the validity of the self-reporting questionnaire and the Afghan symptom checklist: dysphoria, aggression, and gender in

transcultural assessment of mental health. *BMC Psychiatry; 14*: 206. https://doi.org/10.1186/1471-244X-14-20648

Sexton, K. & Dugas, M. J. (2008). Cognitive avoidance questionnaire, *J Anxiety Disord, 22*, 355-70.

Sloan, D.M. et al., (2018). A brief exposure-based treatment vs CPT for PTSD: A randomized non-inferiority clinical trial. *JAMA Psychiatry, 75*, 233-39.

Sutherland, K., & Bryant, R. A. (2008). Social problem solving and autobiographical memory in posttraumatic stress disorder. *Behaviour Research and Therapy, 46,* 154-161.

Taghavi, M.R. & Alishahi, M., J. (2004). Reliability and validity of the revised children's manifest anxiety scale. *Journal of Psychology, 28*, 342-357

Taheri, E. et al. (2016). Cognitive therapy versus behavioural activation therapy in the treatment of social anxiety disorder. *Journal of Fundamentals of Mental Health, 18*, 294

The Economist, March 2019. What disasters reveal about mental-health care. (https://www.economist.com/international/2019/03/16/what-disasters-reveal-about-mental-health-care)

Torgerson, D. & Torgerson, C. (2008). *Designing randomised trials in health, education and the social sciences: An introduction*; 2008. 10.1057/9780230583993.

Tyrer RA, Fazel M (2014) School and community-based interventions for refugee and asylum seeking children: A systematic review. *PLoS ONE 9(2)*, e89359.Doi:10.1371/journal.pone.0089359

UN Children's Fund (2019). *UNICEF Afghanistan Humanitarian Situation Report*

United Nations Office for the Coordination of Humanitarian Affairs (2017). *2018 Afghanistan Humanitarian Needs Overview.*

UNICEF (2019a). *Afghanistan: Adolescent health and development.*
https://www.unicef.org/afghanistan/adolescent-health-and-development

UNICEF (2019b) *Adolescents overview*. https://data.unicef.org/topic/adolescents/overview/

United Nations High Commissioner for Refugees (2019). *Afghanistan*. https://www.unhcr.org/en-au/afghanistan.html

United Nations High Commissioner for Refugees (2020). Operation Portal Refugee Situations: Afghanistan

Werner-Seidler, A, et al., Raes, F., Jobson, L., ..(2018). A cluster RCT comparing group MEST to group psychoeducation and supportive counselling in the treatment of recurrent depression. *BRAT, 105*, 1-9.

Williams, J. M. G., Barnhofer, T., Crane, C., Hermans, D., Raes, F., Watkins, E., & Dalgleish, T.

(2007). Autobiographical memory specificity and emotional disorder. *Psychological Bulletin, 133*,122–148.doi: 10.1037/0033-2909.133.1.122

World Health Organization (2019). *Adolescent Health and Development.* (http://www.searo.who.int/entity/child_adolescent/topics/adolescent_health/en/)

Yazdi, Z., Sadeghniiat-Haghighi, K., Zohal, M. A., & Elmizadeh, K. (2012). Validity and reliability of the Iranian version of the insomnia severity index. *The Malaysian journal of medical sciences: MJMS, 19(4),* 31–36.

Zia, M. S., Afkhami, E., Tavakoli, M., Neshat-Doost, H., & Jobson, L. (in press). A Brief Clinical Report Documenting a Novel Therapeutic Technique (Memory Specificity Training) for Depression: A summary of two preliminary randomized controlled trials. *Behaviour and Cognitive Psychotherapy*
