# Supplementary material for: Effect of the Memory Training for Recovery–Adolescent Intervention vs Treatment as Usual on Psychiatric Symptoms Among Adolescent Girls in Afghanistan: A Randomized Clinical Trial
Source: JAMA Netw Open. 2023 Mar 30;6(3):e236086. doi: 10.1001/jamanetworkopen.2023.6086 (PMC10064255; doi:10.1001/jamanetworkopen.2023.6086)
Supplement: Supplement 2. — eMethods. eResults. eTable. Summary of Baseline Data for Those Who Dropped Out, Those Excluded Due to Adverse Events, and Those Who Completed METRA and TAU eFigure 1. Marginal Means for Anxiety Symptoms by Treatment Group and Follow-up Period eFigure 2. Marginal Means for Afghan-Cultural Distress Symptoms by Treatment Group and Follow-up Period eFigure 3. Marginal Means for Psychiatric Difficulties (Strengths and Difficulties Questionnaire) by Treatment Group and Follow-up Period [file jamanetwopen-e236086-s002.pdf]

Supplemental Online Content

Ahmadi SJ, Jobson L, Musavi Z, et al. Effect of the Memory Training for Recovery–Adolescent intervention vs treatment as usual on psychiatric symptoms among adolescent girls in Afghanistan: a randomized clinical trial. *JAMA Netw Open.* 2023;6(3):e236086. doi:10.1001/jamanetworkopen.2023.6086

eMethods.

eResults.

eTable. Summary of Baseline Data for Those Who Dropped Out, Those Excluded Due to Adverse Events, and Those Who Completed METRA and TAU

eFigure 1. Marginal Means for Anxiety Symptoms by Treatment Group and Follow-up Period

eFigure 2. Marginal Means for Afghan-Cultural Distress Symptoms by Treatment Group and Follow-up Period

eFigure 3. Marginal Means for Psychiatric Difficulties (Strengths and Difficulties Questionnaire) by Treatment Group and Follow-up Period

This supplemental material has been provided by the authors to give readers additional information about their work.

© 2023 Ahmadi SJ et al. *JAMA Network Open.*

eMethods.

Secondary Outcome Measures

Revised Children's Manifest Anxiety Scale (RCMAS)[39]. The RCMAS assessed anxiety. It contains 37 items (28 anxiety items, 9 social desirability items) and scores on the anxiety items were totaled to give a total anxiety score, with higher scores indicating worse symptoms of anxiety. The RCMAS has good psychometric properties[39] and has been used with Afghan youth[15]. Internal consistency was good (McDonald's Omega=.92).

Strengths and Difficulties Questionnaire (SDQ)[40]. The SDQ is a 25-item self-report questionnaire that screens adolescents for psychiatric difficulties (emotional symptoms, conduct problems, hyperactivity/inattention, peer relationship problems) and strengths (prosocial behavior) over the past six months[41]. The SDQ has been used with Afghan youth[10]. Internal consistency was good (McDonald's Omega>.77).

The Afghan Symptom Checklist (ASCL)[41]. The ASCL is a culturally-grounded assessment of psychosocial wellbeing in Afghanistan[41,42]. It contains 22 items and captures several Afghan idioms of distress, including asabi (an anxiety-like state) and jigar khun (a form of sadness)[41,42]. It has good psychometric properties[41,42] and has been used with Afghan youth[12]. Internal consistency was good (McDonald's Omega=.93).

© 2023 Ahmadi SJ et al. *JAMA Network Open.*

eResults

Post-Module 1

At Post-Module 1, when compared to baseline, the METRA group had a 6.31-point (95%CI -8.74,-3.89) decrease in PTSD symptoms, while the TAU group had a 1.41-point (95%CI -3.73,0.91) decrease (p<.01). The METRA group had a 4.13-point (95%CI -5.73,-2.53) decrease in depression, while the TAU group had a 1.44-point (95%CI 0.24,2.65) increase (p<.001). The METRA group had a 3.22-point (95%CI -4.47,-1.97) decrease in anxiety symptoms, while the TAU group had a 0.11-point (95%CI -1.33,1.12) decrease (p<.001). The METRA group had a 11.94-point (95%CI -15.65,-8.23) decrease in Afghan-specific symptoms, while the TAU group had a 2.00-point (95%CI -2.73,6.73) increase (p<.001). Regarding general psychiatric difficulties (SDQ), the METRA had a 1.59-point (95%CI -2.75,-0.42) decrease, while the TAU group had a 1.00-point (95%CI -0.08,2.08) increase (p=.001).

Sensitivity Analysis

GEE indicated that for both PTSD and depression symptoms the group over time interactions were significant. At post-intervention, the METRA group had a 12.20-point (95%CI -14.90,-9.50) decrease in PTSD symptoms from baseline, while the TAU group had a 3.32-point (95%CI -5.71,-0.74) decrease (p< .001). The METRA group had a 4.63-point (95%CI -6.05,-3.20) decrease in depression symptoms from baseline, while the TAU group had a 0.42-point (95%CI -0.80,1.65) increase (p<.001).

At Post-Intervention, the METRA group had a 3.28-point (95%CI -4.40,-2.15) decrease in anxiety symptoms from baseline, while the TAU group had a 0.27-point (95%CI -0.88,1.41) increase (p<.001). The METRA group had a 12.03-point (95%CI -15.32, -8.743 decrease in Afghan-cultural distress symptoms, while the TAU group had a 1.64-point (95%CI -6.34,3.05) decrease (p<.001). The METRA group had a 3.16-point (95%CI -4.25,-2.07) decrease in psychiatric difficulties, while the TAU group had a <.001 (95%CI -1.11,1.11) decrease (p< .001).

At three-month follow-up, compared to baseline, the METRA group had an 13.13-point (95%CI -16.03,-10.22) decrease in PTSD symptoms (TAU=6.09-point, 95%CI -8.93,-3.25, decrease), 4.39-point (95%CI -5.78,-3.00) decrease in depression symptoms (TAU= 0.49-point, 95%CI -2.41,1.43, decrease), 3.70-point (95%CI -5.00,-2.40) decrease in anxiety (TAU= 0.76-point, 95%CI -2.05,0.54, decrease), 10.43-point (95%CI -13.32,-7.53) decrease in Afghan-cultural distress symptoms (TAU=1.96-point, 95%CI -6.34,2.43, decrease), and

© 2023 Ahmadi SJ et al. *JAMA Network Open.*

3.86-point (95%CI -5.30,-2.42) decrease in psychiatric difficulties (TAU=0.31-point, 95%CI -1.04,1.66, increase). All differences in group over time were significant (all p <.01).

© 2023 Ahmadi SJ et al. *JAMA Network Open.*

eTable. Summary of Baseline Data for Those Who Dropped Out, Those Excluded Due to Adverse Events, and Those who Completed METRA and TAU.

**Variable**	**METRA Group – Dropped Out (*n*=18)**	**TAU Group- Dropped Out (*n*=4)**	**METRA Group- Adverse Events (*n*=7)**	**METRA Group- Completed METRA Intervention (*n*=55)**	**TAU Group- Completed TAU Intervention (*n*=41)**
Age -years	16.67 (1.72)	16.75 (3.86)	16.29 (1.70)	15.87 (2.04)	15.65 (1.85)
School Year (7:8:9:10:11:12)	3:1:6:4:4	1:0:0:0:3	1:1:1:3:1	6:11:8:8:15:7	7:9:6:7:7:4
Number of Family Members	8.41 (2.35)	5.67 (1.53)	10.00 (2.83)	8.28 (1.92)	8.59 (2.22)
PTSD Symptoms	40.83 (7.11)	39.25 (8.81)	43.43 (9.89)	42.09 (8.81)	44.90 (7.44)
Depression Symptoms	17.33 (5.36)	13.50 (7.05)	18.86 (5.90)	17.76 (5.59)	17.54 (5.50)
Anxiety Symptoms	24.72 (3.66)	23.75 (3.30)	29.14 (3.39)	26.31 (4.18)	26.46 (3.70)
Afghan Cultural-Distress Symptoms	48.33 (16.66)	43.75 (13.79)	55.57 (17.23)	49.22 (15.26)	49.90 (16.63)
Psychiatric Symptoms	18.28 (3.54)	13.00 (2.94)	19.00 (4.97)	18.58 (5.94)	19.32 (4.63)

© 2023 Ahmadi SJ et al. *JAMA Network Open.*

eFigure 1. Marginal Means for Anxiety Symptoms by Treatment Group and Follow-up Period

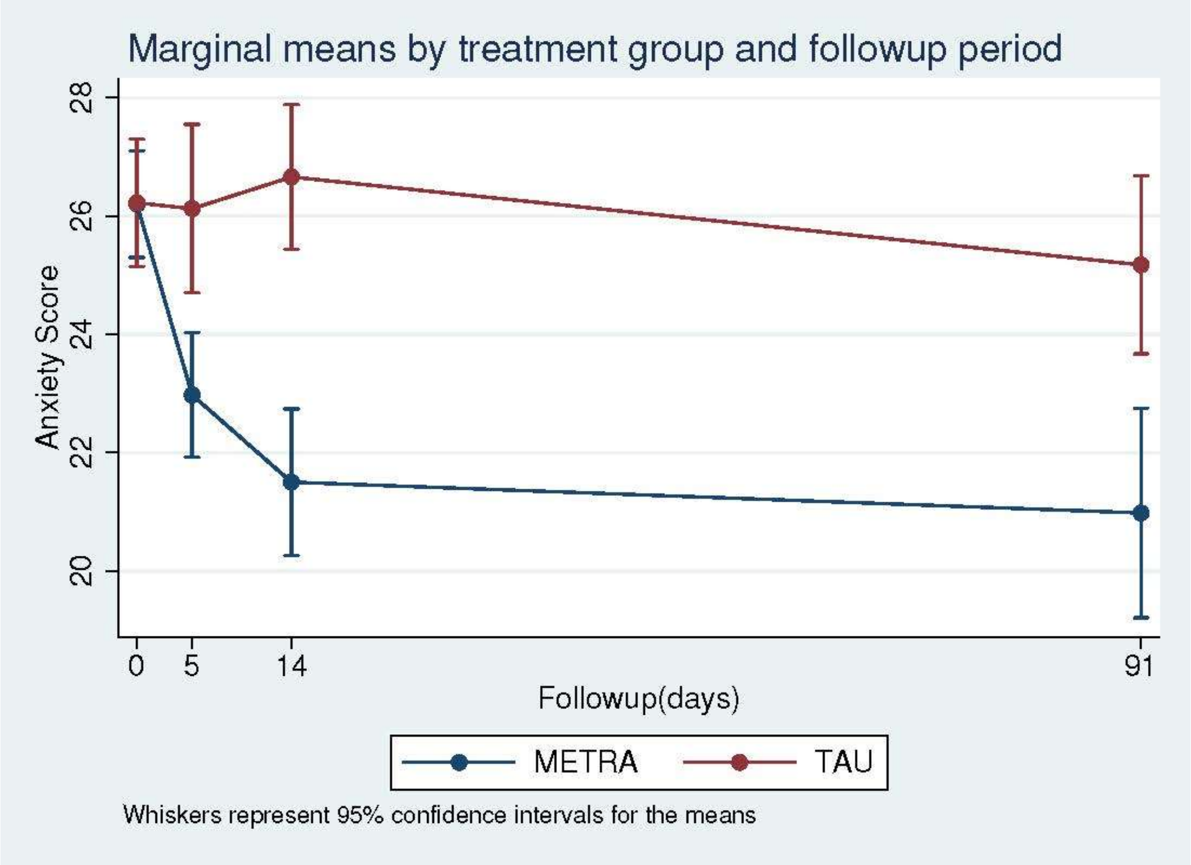

© 2023 Ahmadi SJ et al. *JAMA Network Open.*

eFigure 2. Marginal Means for Afghan-Cultural Distress Symptoms by Treatment Group and Follow-up Period

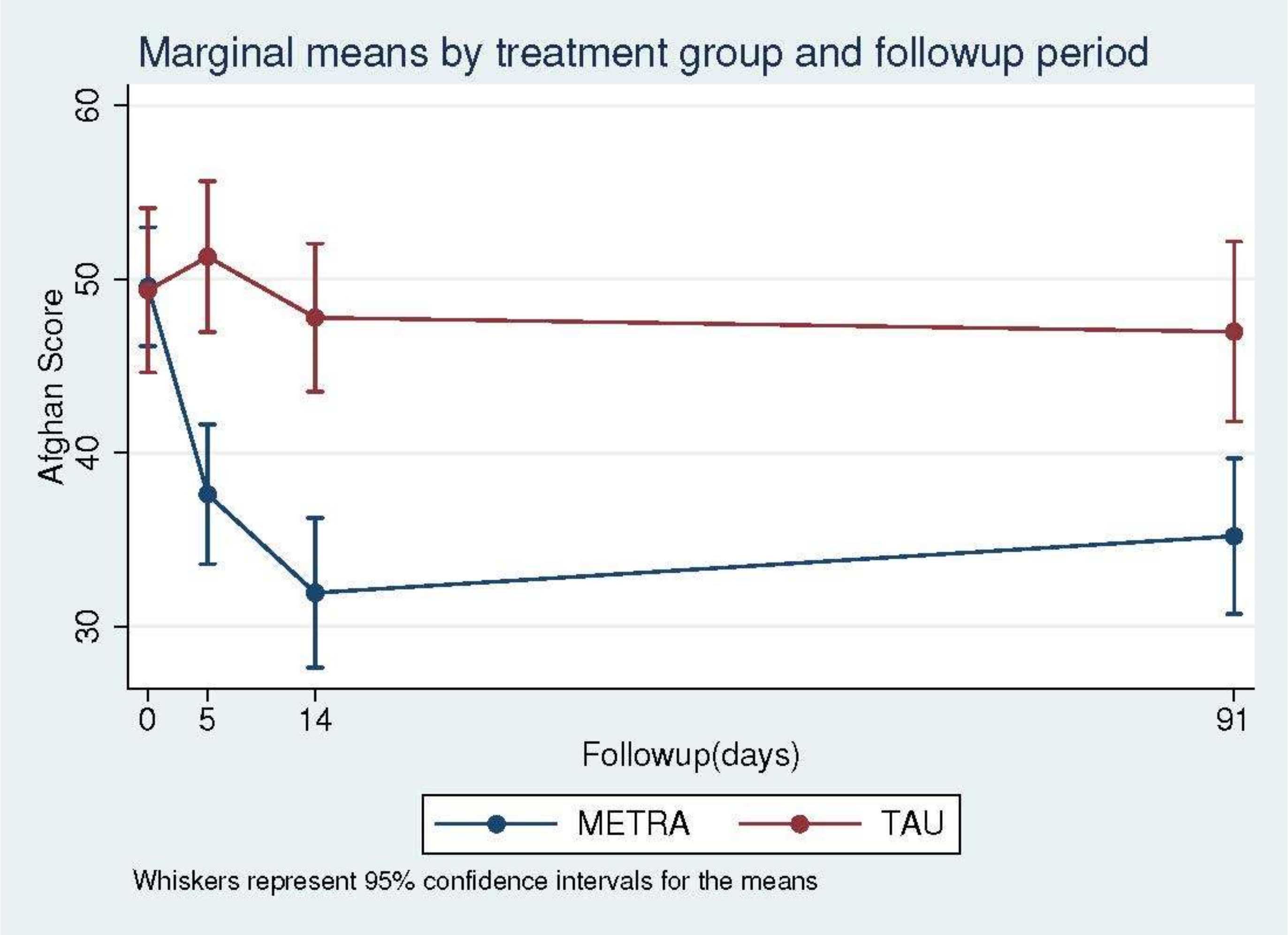

© 2023 Ahmadi SJ et al. *JAMA Network Open.*

eFigure 3. Marginal Means for Psychiatric Difficulties (Strengths and Difficulties Questionnaire) by Treatment Group and Follow-up Period

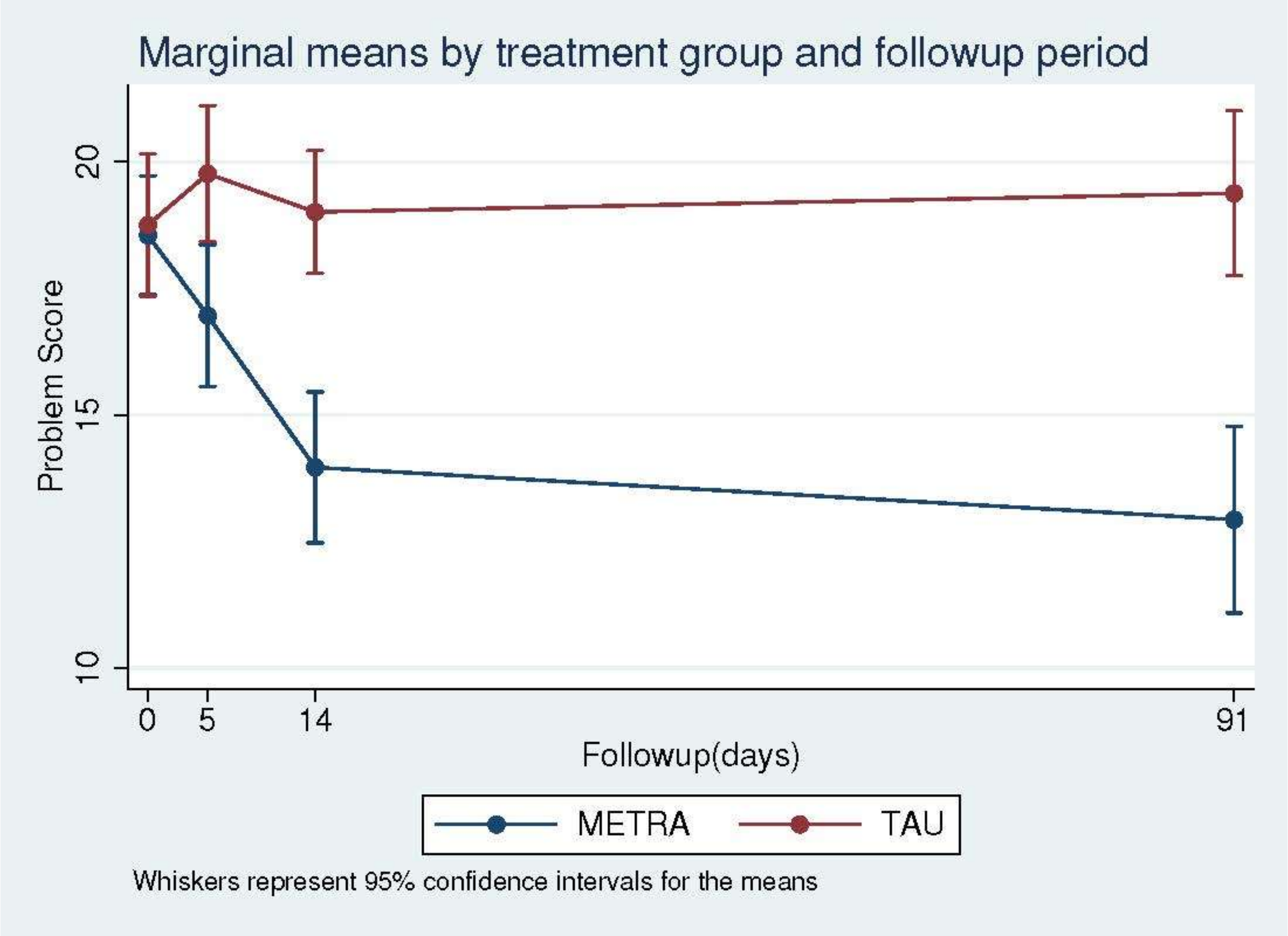

© 2023 Ahmadi SJ et al. *JAMA Network Open.*
