# Supplementary material for: Effect of the Memory Training for Recovery–Adolescent Intervention vs Treatment as Usual on Psychiatric Symptoms Among Adolescent Girls in Afghanistan: A Randomized Clinical Trial
Source: JAMA Netw Open. 2023 Mar 30;6(3):e236086. doi: 10.1001/jamanetworkopen.2023.6086 (PMC10064255; doi:10.1001/jamanetworkopen.2023.6086)
Supplement: Supplement 3. — Data Sharing Statement [file jamanetwopen-e236086-s003.pdf]

Data Sharing Statement

Ahmadi. Effect of the Memory Training for Recovery-Adolescent Intervention vs Treatment as Usual on Psychiatric Symptoms Among Adolescent Girls in Afghanistan. *JAMA Netw Open*. Published March 30, 2023. doi:10.1001/jamanetworkopen.2023.6086

Data
Data available: Yes
Data types: Deidentified participant data
How to access data: Data will be made available upon request by contacting the corresponding authors.
When available: With publication

Supporting Documents
Document types: Other (please specify)
Additional Information: Trial protocol and treatment manual
How to access documents: Open Science Framework (https://osf.io/tqfgr/)
When available: With publication

Additional Information
Who can access the data: researchers whose proposed use of the data has been approved (due to security issues associated with Afghanistan)
Types of analyses: any purpose
Mechanisms of data availability: after approval of a proposal (due to security issues in Afghanistan)
